# A moving-window bayesian network model for assessing systemic risk in financial markets

**DOI:** 10.1371/journal.pone.0279888

**Published:** 2023-01-20

**Authors:** Lupe S. H. Chan, Amanda M. Y. Chu, Mike K. P. So

**Affiliations:** 1 Department of Information Systems, Business Statistics and Operations Management, The Hong Kong University of Science and Technology, Clear Water Bay, Hong Kong; 2 Department of Social Sciences, The Education University of Hong Kong, Tai Po, Hong Kong; Beihang University, CHINA

## Abstract

Systemic risk refers to the uncertainty that arises due to the breakdown of a financial system. The concept of “too connected to fail” suggests that network connectedness plays an important role in measuring systemic risk. In this paper, we first recover a time series of Bayesian networks for stock returns, which allow the direction of links among stock returns to be formed with Markov properties in directed graphs. We rank the stocks in the time series of Bayesian networks based on the topological orders of the stocks in the learned Bayesian networks and develop an order distance, a new measure with which to assess the changes in the topological orders of the stocks. In an empirical study using stock data from the Hang Seng Index in Hong Kong and the Dow Jones Industrial Average, we use the order distance to predict the extreme absolute return, which is a proxy of extreme market risks, or a signal of systemic risks, using the LASSO regression model. Our results indicate that the network statistics of the time series of Bayesian networks and the order distance substantially improve the predictability of extreme absolute returns and provide insights into the assessment of systemic risk.

## Introduction

Systemic risk refers to a breakdown of a financial system, which leads to a severe economic downturn, caused by connections within the financial system or natural disasters. Systemic risk is extremely harmful, since it may trigger cascading failures and losses [1, 2]. Cascading failure refers to a process in which the failure of a few parts in an interconnected system triggers the failure of the other parts. If a financial system is tightly connected, the failure of a small part can eventually lead to a huge turnover of the whole system. This leads to the concept of “too connected to fail” [3]. Several works have documented higher level of network connectedness during the financial crises [4, 5]. Examples include the financial crisis of 2007–2008 due to the default of an investment bank and the close links between the financial systems [2, 6], the Chinese stock market turbulence in 2015 due to the inflation of the stock market bubble [7, 8], and the sharply increased systemic risk during the COVID-19 pandemic [9]. Various approaches to measuring systemic risks have been studied, including the marginal expected shortfall [10] and the conditional value-at-risk [11]. These measures take correlations into account, whereas a correlation alone is not adequate for capturing connectedness, since it does not consider the interactions or the systemic importance in a financial system [12]. Network analysis is a much more comprehensive way of evaluating connectedness than value-at-risk. A network refers to a graph that represents a relationship using connections among a group of objects, and a financial network is a network with stocks as the objects. The group of objects and the connections are called nodes and edges, respectively, in the context of network analysis. By analyzing the high level of network connectedness in a financial network [13], we may be alerted before the market disturbances spread, which leads to a high level of volatility in a market. Volatility can be considered as a trigger or the cause of a crisis, and volatility can serve as a kind of precautionary signal for systemic risks. The best we can do is to avoid excessive volatility [14]. Throughout the paper, we aim to develop a measure to improve the prediction performance of the extreme financial risk, referred to as the order distance. We also propose the use of a rolling-window method in regard to learning a time series of Bayesian networks, in order to learn changes in the topological properties of the financial market, especially the order distance. We showcase how the topological features of time series of Bayesian networks can help assess financial risk using two sets of data from the Hang Seng Index (HSI) and the Dow Jones Industrial Average (DJIA) Index.

Rolling-window methods have been applied to financial risk management in previous studies. For example, [15] uses a rolling-window method to learn a time series of undirected networks for a stochastic actor-oriented model; [16] proposes a textual modeling method using a rolling-window scheme in a regression model, to predict a market volatility proxy based on the GARCH residuals; [17] studies the effect of the COVID-19 pandemic on financial market connectedness and systemic risk using rolling-window Granger-causality tests; and [18] uses a rolling-window method and a combined method with the topic modeling and network analysis to assess systemic risk in financial markets. Network analysis has been applied to risk assessment [19], pandemic risk management [20–22] and financial risk management [13, 18, 23, 24]. To quantify extraordinary financial risk due to financial connectedness or financial contagion, a common application of network analysis involves systemic risk modeling [25–27]. There are recent examples measuring financial companies’ contributions to systemic risk through the financial networks built using the tail risk of asset returns [28], conducted in order to study the impacts of the COVID-19 pandemic on the financial networks, based on partial correlations of the stock returns [13]; to detect early signals of financial contagion from financial networks, based on partial correlations of stock returns, and pandemic networks, based on the daily newly confirmed cases [17]; and to assess systemic risk in financial networks in terms of dynamic topic networks, based on topic similarities in the news [18]. The above studies are categorized as undirected graph applications, where edges in their networks have no direction. In this paper, we consider the use of a rolling-window Bayesian network model for assessing systemic risks in financial markets.

A Bayesian network is a popular probabilistic graphical model that represents a set of variables and their conditional dependence using a directed acyclic graph (DAG). A DAG is a graph containing only directed connections; it does not contain a cycle. The nodes in any DAG can be ranked in a topological order [29]; that is, we can tell which nodes are more important from the DAG. This feature effectively fits systemic modeling, in that we can measure the relative importance of the stocks, such that the stocks ranked at the front of the topological order can be associated with the sources of the market disturbances, and the topological order itself can be used as a list of directions regarding the flow of the market disturbances. Bayesian networks are often used to represent causal relationships; given an event occurring, we can calculate the probability of the consequences. Bayesian networks have been used in many real-world applications, including diagnosis and forecasting [30, 31], and constitute an effective modeling approach in risk management [32], such as in modeling systemic risk using credit default swap data [33]; in detecting variables that affect the buy-sell signal for the S&P 500 index, including price-to-earnings ratio and price-to-sales ratio [34]; in credit risk modeling [35]; in modeling operational risk [36]; and in using DBN to generate early warning signals in systemic banking crises [37].

Note that, although we analyze a time series of Bayesian networks, our proposed methodology does not involve standard dynamic Bayesian network modeling. A dynamic Bayesian network models the dependency of variables over adjacent time steps. For example, the nodes of a sequences of Bayesian networks are assumed to follow homogeneous first-order Markov processes [38]; that is, the nodes at time *t* are dependent only on the nodes at time *t*−1. Another example is a framework specifically modeling the evolution of the dependencies of a sequence of Bayesian networks [39]. We do not model the evolution of the dependencies, but we do try to extract information from a time series of Bayesian network using a rolling-window method.

We propose the order distance, an indicator that measures the change in the topological orders of the Bayesian networks, which are learned using the rolling-window method, on two consecutive days, using MCMC structural learning algorithms [40, 41]. We aim to use the order distance to improve the volatility prediction for the assessment of systemic risk in a financial market. We provide empirical evidence that a high level of change in the topological order is often accompanied by a high volatility. The empirical evidence is discussed in the section on order distance, and the detailed results are in S3 Appendix in S1 File. The order distance is used as predictor in a LASSO prediction model. Another indicator, the modified network density, is also included in the predictive model, treated as a predictor or a control variable. The Granger-causality tests are significant in regard to quite large portions of trading days, supporting the idea that the two risk indicators are useful in predicting the absolute returns. We illustrate the use of DBN and the topological order to help predict extreme market changes, and thus provide insights into assessing systemic risk. We will show that the two indicators are useful for improving the prediction performance of the volatility using a LASSO regression prediction model. It supports that the order distance contains useful information for predicting volatility. Also note that, the proposed order distance is not restricted to being used as a predictor in our LASSO prediction model, but it could be applied to any other volatility model as a predictor.

**Fig 1:**
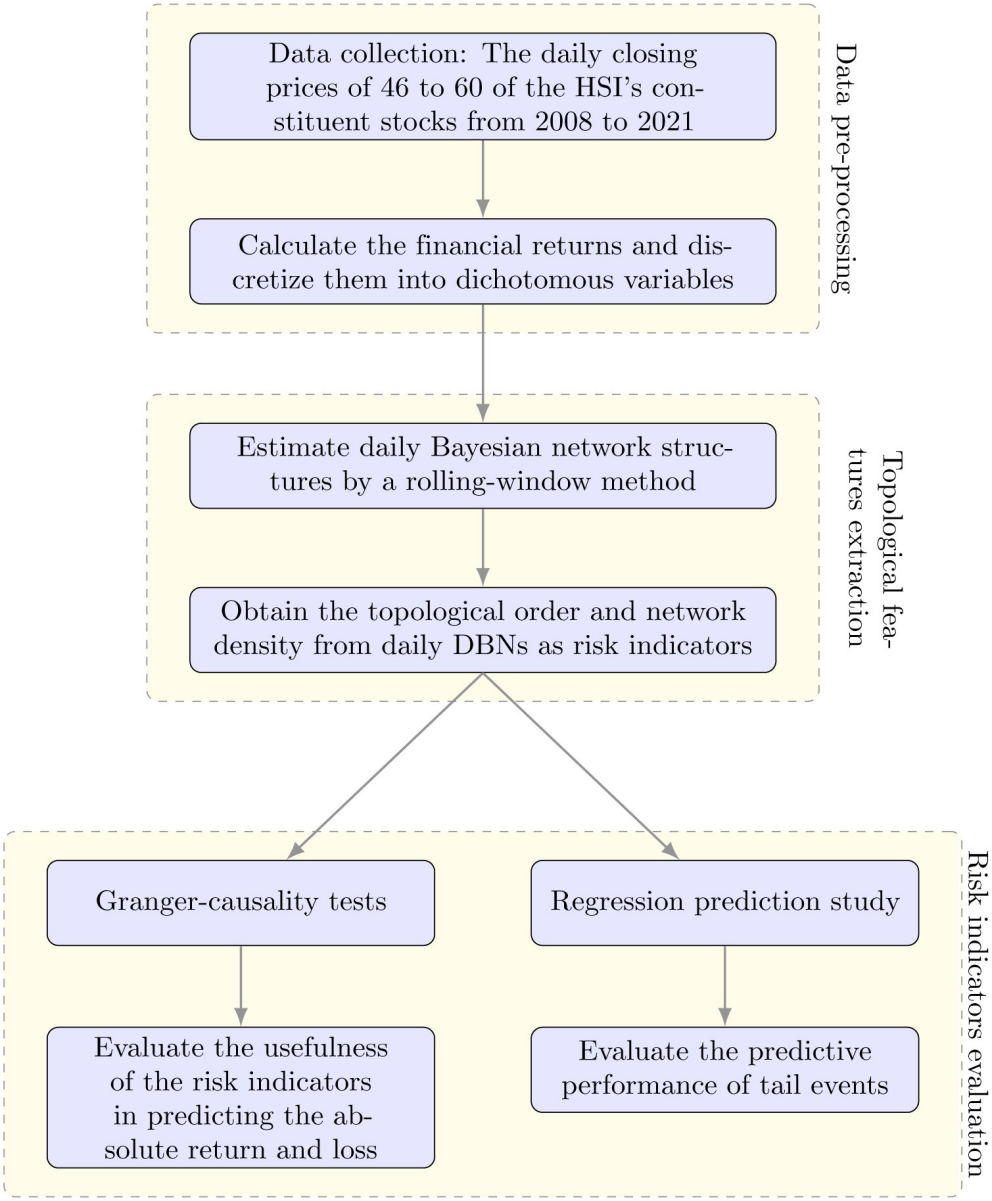
An overview of the methodology flow in our study.

Specifically, an overview of the methodology of this study is shown in Fig 1. In this paper, we use the financial returns of 46 to 60 constituent stocks in the Hang Seng Index of Hong Kong from 2008 to 2021 (3,404 trading days) to estimate daily Bayesian network structures using a rolling-window method. The numbers of the HSI’s constituent stocks were increased from 46 to 60 over the study period. The returns were discretized into dichotomous variables (0: downward movement; 1: upward movement) to make modeling more straightforward. We combine several structural learning algorithms to enhance the computational efficiency. The topological order and modified network density are then obtained from daily DBNs as risk indicators, measuring topological features derived from the inherent Markov dependence in Bayesian networks on each trading day. We expect that these topological features to provide useful information on market risks, especially under adverse and extreme market conditions. These two risk indicators were adopted to predict a proxy of the volatility, the absolute return of the HSI. Using the absolute return as a proxy of volatility is a well-established method [42–44]. This proxy has been widely used as a measure of market risk in the literature [16, 45]. We also use the loss, defined as the negative part of the return of the HSI, as a proxy of market losses. To investigate the effectiveness of the two risk indicators, Granger-causality tests were conducted using the absolute return and the loss as responses to see when the risk indicators are useful to predict the absolute return or the loss. The predictive performance was also reported, with particular attention paid to tail events. The above methodology is also applied to the Dow Jones Industrial Average Index in order to test the predictive performance of the order distance in different stock markets.

The Methodology Section introduces the concepts related to Bayesian networks, including the network statistics, network density, and topological orders. The details of the proposed order distance are also included in the Methodology Section. The Rolling-window Bayesian Network Modeling Section contains the details of the data used in the empirical study in this paper, the procedures involved in the structural learning of a time series of Bayesian networks, and the details of the methods used to evaluate the usefulness of the order distance (including the Granger-causality tests and a LASSO regression prediction model). The Main Results Section provides an illustration of the learned financial networks and their summary statistics, the Granger-causality tests results, and the prediction performances of the rolling-window prediction study. The Discussion Section explains the results in the Main Results Section and their implications. A discussion of the choice of the parameters is also included. A conclusion is also provided in the Discussion Section.

## Methodology

### Bayesian networks

In this paper, we adopt a moving-window scheme in regard to Bayesian networks for modeling the dependence of financial returns over time. A Bayesian network (BN) is a graphical model that can be used to specifying the joint probability distributions of a set of variables [46]. More specifically, BNs can represent conditional dependence among variables using a directed acyclic graph (i.e., a directed graph that does not contain any cycles). The graphical representation of a BN is called a network, a graph, or a structure. A variable in a BN is called a node, and a directed connection to or from a node is called an arc or a directed edge. Let *X*_1_, …, *X*_*n*_ be *n* variables in a BN. We say that an edge *X*_*i*_ → *X*_*j*_ is in the BN if there exists an edge linking *X*_*i*_ to *X*_*j*_ in the BN. When we have the edge *X*_*i*_ → *X*_*j*_ in the BN, *X*_*i*_ is a said to be a parent of *X*_*j*_ and *X*_*j*_ is said to be a child of *X*_*i*_. When there is a path *X*_*i*_ → … → *X*_*j*_ linking *X*_*i*_ to *X*_*j*_ by a sequence of edges, *X*_*j*_ is said to be a descendant of *X*_*i*_. By definition, a child of a node *X*_*i*_ is also a descendant of *X*_*i*_. The parent set of a variable *X*_*i*_ is denoted by pa(*X*_*i*_), which contains all parents of *X*_*i*_. A network is said to contain a cycle if there exists a node *X* and a sequence of nodes *Y*_1_, …, *Y*_*j*_ such that the path *X* → *Y*_1_ → *Y*_2_ → … → *Y*_*j*_ → *X* is in the BN. The structure of any BN has to be a DAG, meaning that a BN does not contain any cycles. A BN satisfies the Markov assumption, which states that conditional on the parent set, a node is independent of other variables except its descendants. From the Markov assumption, the joint density function of the BN of *n* variables *X*_1_, *X*_2_, …, *X*_*n*_ can be factorized into

$$\begin{array}{c} P\left(X_{1},X_{2},\ldots ,X_{n}\right)=\prod_{i=1}^{n}P\left(X_{i}|\text{pa}\left(X_{i}\right)\right). \end{array}$$ (1)

**Fig 2:**
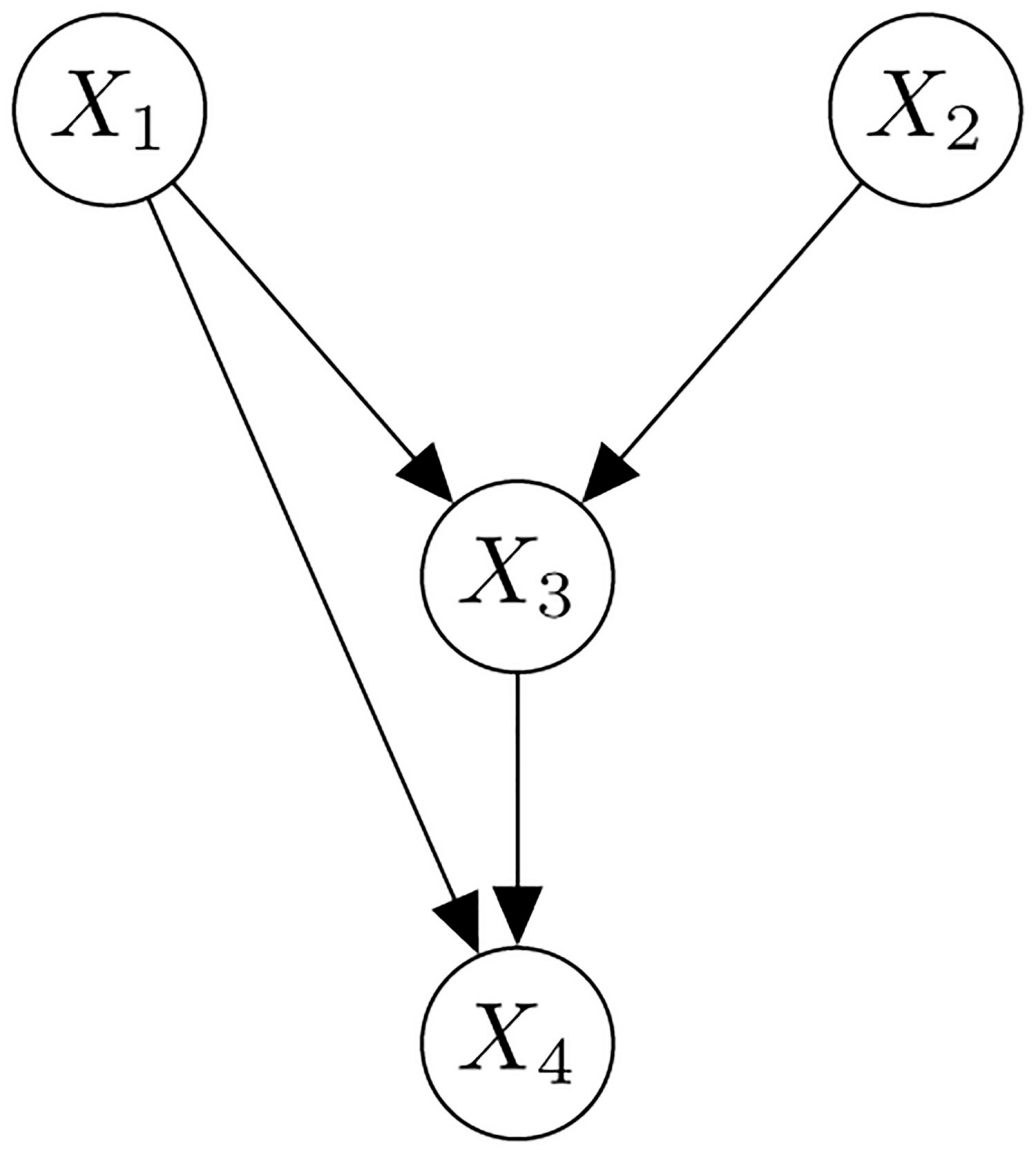
An example of a Bayesian network of four variables, *X*_1_, *X*_2_, *X*_3_, and *X*_4_.

Fig 2 example gives an example of a BN consisting four variables: *X*_1_, *X*_2_, *X*_3_, and *X*_4_. The arrows between variables represent their edges, and the edges specify the dependence structures among the variables. For example, *X*_4_ depends on both *X*_3_ and *X*_1_, and *X*_1_ and *X*_2_ are the root nodes (i.e., nodes with no incoming edge). The joint probability distribution of the four variables can be factorized based on the dependence structure specified in Fig 2 example:

$$\begin{array}{c} P\left(X_{1},X_{2},X_{3},X_{4}\right)=P\left(X_{4}|X_{3},X_{1}\right)P\left(X_{3}|X_{1},X_{2}\right)P\left(X_{2}\right)P\left(X_{1}\right). \end{array}$$ (2)

The factorization in Eq (1) greatly simplifies the complex connectedness among variables and forms a score for the BN structural learning [29].

### Network statistics

In general, Bayesian networks can be time-varying. In the discussion below, we denote a BN at time *t* by $G_{t}$, which is defined by its set of nodes and its set of edges at time *t*. To efficiently summarize the statistical properties of $G_{t}$, the following two summary statistics are used. The first one is the network density, and the second one is the topological order of the variables.

#### Network density

The network density measures the connectedness of a network. Intuitively, the more connections or more edges a BN has, the higher the network density will be. The network density at time *t* is defined as

$$\begin{array}{c} ND\left(G_{t}\right)=\frac{2e_{t}}{v_{t}\left(v_{t}-1\right)}, \end{array}$$ (3)

where *v*_*t*_ and *e*_*t*_ are, respectively, the number of nodes and the number of edges of the network $G_{t}$. It is the ratio of the number of edges in $G_{t}$ to the maximum possible number of edges, (vt2)=vt(vt-1)/2, implying that $ND(G_{t})$ lies between 0 and 1. As edges in a BN represent directed links among variables, $ND(G_{t})$, which gives the proportion of edges in the BN, quantifies the complexity of the BN to represent hidden conditional dependence structures among variables. In the structural learning of Bayesian networks, we often impose a restriction on the maximum number of parents for each node for the sake of computational efficiency. Under this restriction, the maximum possible number of edges is much smaller than (vt2). Suppose that the maximum number of parents of each node is *M* (*M* ≤ *v*_*t*_). It can be shown that with the restriction, the maximum possible number of edges in a BN is *v*_*t*_*M*−*M*(*M* + 1)/2. More details of this maximum number of edges are provided in the S2 Appendix in S1 File. We define the modified network density with the maximum parent size *M* as

$$\begin{array}{c} MND\left(G_{t};M\right)=\frac{e_{t}}{v_{t}M-M\left(M+1\right)/2}. \end{array}$$ (4)

The network density in Eq (4) extends that in Eq (3). For the special case of *M* = *v*_*t*_−1, in which there is no restriction on the maximum number of parents, $MND(G_{t};v_{t}-1)$ is reduced to $ND(G_{t})$. In the empirical study, we take *M* = 13, since the computation will be very slow when we choose a larger *M*. We will see how Eq (4) can be used to quantify systemic risk from the connectedness of the stocks in the empirical study.

#### Topological orders

Bayesian networks are also known as causal networks because the network configuration may give us information on the “cause-and-effect” interpretation from a probabilistic perspective. For example, the factorization in Eq (2) for the BN in Fig 2 example gives us an idea of which variables are the “causes” and/or “effects” of the other variables. In the joint distribution in Eq (2), *X*_1_ appears in *P*(*X*_1_), *P*(*X*_3_|*X*_1_, *X*_2_), and *P*(*X*_4_|*X*_3_, *X*_1_). We can interpret that *X*_1_ as a “cause” of *X*_3_ but not vice versa because *X*_1_ appears in the conditional distribution of *X*_3_ as a given variable in the factorization. Similarly, *X*_1_ and *X*_3_ can be regarded as “causes” of *X*_4_ according to the network structure in Fig 2 example. If we take the potential cause-and-effect structure into consideration, we can say a “cause” variable is lower than an “effect” variable. We can then talk about topological order as a topological feature of a BN.

A topological order of a BN defined in [29] refers to the ranking of the variables in the network. In a Bayesian network, if *X*_*i*_ precedes *X*_*j*_ in the topological order, then an edge from *X*_*j*_ to *X*_*i*_ is not allowed or *X*_*j*_ is not a parent of *X*_*i*_. For example, in Fig 2 example, one possible topological order is (*X*_2_, *X*_1_, *X*_3_, *X*_4_) according to the rule stated above. Let *T*(*X*_*i*_) be the position of *X*_*i*_ in a topological order. Then, the topological order (*X*_2_, *X*_1_, *X*_3_, *X*_4_) in Fig 2 example corresponds to *T*(*X*_1_) = 2, *T*(*X*_2_) = 1, *T*(*X*_3_) = 3 and *T*(*X*_4_) = 4. The topological order (*X*_1_, *X*_2_, *X*_3_, *X*_4_) is also allowed since neither *X*_1_ nor *X*_2_ is a parent of each other. For this topological order, we have *T*(*X*_1_) = 1, *T*(*X*_2_) = 2, *T*(*X*_3_) = 3 and *T*(*X*_4_) = 4. As there are multiple topological orders corresponding to a given BN, a fairer way to rank the variables is to take the median of all possible topological orders. It can be very computationally intensive to list out all possible topological orders when the network size (i.e., the number of nodes *v*_*t*_) is large. Instead of identifying all topological orders of a Bayesian network at time *t*, $G_{t}$, an alternative method is to sample a subset of topological orders from $O_{G_{t}}$, the space of all possible topological orders corresponding to $G_{t}$. Then, we can estimate $M_{t}\left(X_{i}\right)=median\left\{T_{t}\left(X_{i}\right):\left(T_{t}\left(X_{1}\right),...,T_{t}\left(X_{v_{t}}\right)\right)\in O_{G_{t}}\right\}$ using the sampled topological orders. We use the Kahn’s algorithm [47] to help generate samples from $O_{G_{t}}$. The Kahn’s algorithm is a node sorting algorithm. We need to input an initial topological order of the nodes into this algorithm, then the algorithm will start to sort the nodes based on the initial topological order. For each initial topological order, the algorithm returns a topological order that is compatible with $G_{t}$. To generate samples of topological orders from $O_{G_{t}}$, we can input different randomly generated initial topological orders to the algorithm. There are *v*_*t*_! ways to input the initial topological orders. In the empirical study, we will randomly pick *K* = 100 initial topological orders to input into the algorithm for obtaining samples of topological orders for each $G_{t}$ and to estimate *M*_*t*_(*X*_*i*_) using the samples.

To visualize and compare the topological order of the nodes on different days, since the numbers of nodes can change over time, the range of *M*_*t*_(*X*_*i*_) can be different. To facilitate the comparison, a relative topological order of the network $G_{t}$ is defined:

$$\begin{array}{c} RM_{t}\left(X_{i}\right)=\frac{v_{t}-M_{t}\left(X_{i}\right)}{v_{t}-1}. \end{array}$$ (5)

Note that, if *M*_*t*_(*X*_*i*_) = 1 (i.e., at the head of the topological order), then *RM*_*t*_(*X*_*i*_) = 1; if *M*_*t*_(*X*_*i*_) = *v*_*t*_ (i.e., at the tail of the topological order), then *RM*_*t*_(*X*_*i*_) = 0. *RM*_*t*_(*X*_*i*_) always lies between 0 and 1, and a larger *RM*_*t*_(*X*_*i*_) indicates that *X*_*i*_ is relatively near the head of the topological order. As in *M*_*t*_(*X*_*i*_), we can estimate *RM*_*t*_(*X*_*i*_) by using sampled topological orders from $O_{G_{t}}$.

In financial modeling, this relative topological order allows us to quantify the importance of each stock in a Bayesian network at different time points. If a stock is topologically ordered near the front, it can be regarded as the “cause” of the changes in other stocks in the Bayesian network, or as part of the sources of market fluctuation. On the other hand, if a stock is topologically ordered near the tail, the stock’s influence on other stocks’ fluctuation may be relatively smaller. In short, *RM*_*t*_(*X*_*i*_) helps measure the relative importance of the stocks in a BN over time. If *RM*_*t*_(*X*_*i*_) changes substantially in a short amount of time, it may indicate unexpected changes in the market fluctuation, or rapid increase of systemic risk in financial markets.

#### Order distance

To trace the stability of financial markets over time, we propose measuring the changes in the topological orders. The rationale behind this is that the network topology is likely to be stable when the market is “quiet” or the volatility of the market is low. Conversely, the network topology can change substantially when there are severe adverse changes in the market. Therefore, being able to quantify changes in the topological order can help assess financial risk, especially for events leading to extreme market movement, which triggers systemic risk in financial markets. Let *V*_*t*_ be the set of nodes in the Bayesian network at time *t*. Consider the vector of median topological orders, ${\left\{M_{t}\left(X_{i}\right)\right\}}_{i=1,...,v_{t}}$, on two consecutive days: day *t*−1 and day *t* for the stock *X*_*i*_. Since *V*_*t*_ and *V*_*t*−1_ may have different nodes, we only consider the nodes in *V*_*t*−1_∩*V*_*t*_ to define a topological order distance on day *t*. Note that the scale of *M*_*t*_(*X*_*i*_) and *M*_*t*−1_(*X*_*i*_) can also be different, since *M*_*t*_(*X*_*i*_) takes values from 1 to *v*_*t*_. Following the idea of setting portfolio weights in asset allocation, we can define a vector of normalized topological orders on day *t* with respect to the set *V*_*s*_ as

$$\begin{array}{c} {NT}_{t}\left(V_{s}\right)={\left[\frac{v_{t}-M_{t}\left(X_{i}\right)}{\sum_{X_{j}\in V_{s}}[v_{t}-M_{t}\left(X_{j}\right)]}\right]}_{X_{i}\in V_{s}}. \end{array}$$ (6)

By construction, all elements in **NT**_*t*_(*V*_*s*_) lie between 0 and 1 and $\sum_{X_{i}\in V_{s}}NT_{t}\left(X_{i}\right)=1$, where *NT*_*t*_(*X*_*i*_) is an element in **NT**_*t*_(*V*_*s*_). The elements in the vector in Eq (6) are analogous to the portfolio weights for investing in the stocks represented by the nodes in *V*_*s*_, putting more weights on the stocks near the head of the topological orders.

The order distance between two consecutive networks on trading day *t*−1 and *t* is defined as

$$\begin{array}{c} OD_{t}=∥{{NT}_{t}\left(V_{t-1}\right)-{NT}_{t-1}\left(V_{t-1}\right)∥}_{1}, \end{array}$$ (7)

where ∥⋅∥_1_ is the *L*^1^-norm.

We use of *L*^1^-norm to define the order distance because in **NT**_*t*_(⋅), *v*_*t*_ − *M*_*t*_(*X*_*i*_) is normalized by the sum of its elements (which are all positive). This normalization can be regarded as a *L*^1^-normalization. If we choose *L*^*p*^-norms (*p* > 1), the corresponding order distance will tend to decrease when the dimension of **NT**_*t*_(⋅) increases (which is undesirable for a regression predictor).

We use the order distance because we believe that there is a relationship between a high level of volatility and a rapid change in network connectedness, since a volatile market is often unstable. Specifically, we posit that the association between the volatility and order distance is more likely to be positive whenever the volatility is high. We provide empirical evidence of this phenomenon in S3 Appendix in S1 File. We fit moving-window regressions using the absolute daily returns of the Hang Seng Index, a proxy of the volatility, as the responses, and the estimated order distances in the previous trading day as the predictor. The modified network density and the lagged absolute returns are also included as control variables. We obtained the estimates of the coefficients of the order distances on each of the trading days over the study period. We partition the log-transformed absolute returns (log-transformation is conducted because we want to ensure the results can be easily visualized), and count the proportion of positive coefficients in each of the intervals. We found that the proportions are, on average, larger whenever the absolute returns are larger. The same analysis is conducted using the Dow Jones Industrial Average Index; the proportions show a similar phenomenon. The order distance is thus useful for predicting volatility in a period with high levels of volatility, and a high level of volatility is often accompanied by a large order distance. Based on this phenomenon, we will focus on extreme scenarios in this paper. We provide a more detailed discussion of these extreme scenarios in the LASSO Regression Prediction section later in this paper. More details of the above empirical evidence can be found in S3 Appendix in S1 File.

### Rolling-window Bayesian network modeling

#### Data

To demonstrate our proposed Bayesian network method in assessing systemic risk using topological features of the network, we obtain the closing prices of the HSI’s constituent stocks [48] in the Hong Kong stock market and the closing prices of HSI from 2 January 2008 to 4 November 2021 (3,404 trading days). We use the HSI because the Hong Kong Stock Exchange is one of the most representative exchanges in the world [49]. We believe that the Hong Kong stock market is a useful example for studying systemic risk. Moreover, the constituent companies in the HSI represent about 65% of the capitalization of the Hong Kong Stock Exchange [50]. It is important to study the systemic risk of the constituent stocks in the HSI. A time series plot of the HSI is given in Fig 7. The constituent names, stock symbols and their sectors are listed in the S1 Appendix in S1 File. Let *P*_*it*_ and *R*_*it*_ = log*P*_*it*_ − log*P*_*i*(*t*−1)_ be the closing price and log-return, respectively, of the *i*-th stock on day *t*, where day 0 refers to 2 January 2008. The data on 2 January 2008 are used to calculate the log-return on 3 January 2008, so that there are altogether *T* = 3403 returns in our investigation. Since the composition of the HSI is reviewed regularly, both the numbers of constituent stocks and the list of constituent stocks change over time. In the period of the empirical study, the number of the constituent stocks increases gradually from 46 to 60. The construction of the time series of Bayesian networks is based on the HSI’s constituent stocks. Therefore, the number of nodes *v*_*t*_ ranges from 46 to 60 in the empirical study.

In this paper, we focus on dichotomous return variables in the Bayesian network construction though, in general, our methodology can also be applicable to continuous variables. From a practical perspective, it is also easier to model Bayesian networks using discrete data than continuous data. Specifically, we define the return indicator of the *i*-th stock on day *t* by

$$\begin{array}{c} X_{it}=\{\begin{array}{cc} 1 & \;\text{if}\;R_{it}\geq 0\\ 0 & \;\text{if}\;R_{it}<0, \end{array} \end{array}$$ (8)

for *t* = 1, …, *T*, where *t* = 1 refers to 3 January 2008. *X*_*it*_ captures the direction of movement of stock *i* on day *t*. It is expected that learning the topological features of the stocks over time through the time series of Bayesian networks can provide useful information about the risk propagation in financial markets.

#### Structural learning

We adopt a rolling-window approach and use the return indicators *X*_*it*_ to construct a time series of Bayesian networks. Let *D*_*t*_ be the set of return indicator variables of the HSI’s constituent stocks on day *t*. For each day *t*, we use the data sets *D*_(*t*−*w*+1):*t*_ = {*D*_*t*−*w*+1_, …, *D*_*t*_} to construct $G_{t}$, the BN on day *t*, where *w* is referred to as the rolling-window size. Since the number of variables in *D*_*t*_ can be different on different day *t*, the network $G_{t}$ only includes variables that are common in all data sets in *D*_(*t*−*w*+1):*t*_. We employ the Markov chain Monte Carlo (MCMC) structural learning algorithms, including the Structure MCMC algorithm [40] and the Order MCMC algorithm [41] to estimate $G_{t}$ by obtaining the network that maximizes the Bayesian Dirichlet equivalent uniform (BDeu) score [29] using the data set *D*_(*t*−*w*+1):*t*_.

Structural learning refers to a task for learning the network structure of a set of variables from the data. There are mainly two classes of structural learning algorithm: score-based algorithms, which maximizes an objective function, and constraint-based algorithms, which tests for conditional independence structures in the data. MCMC is a score-based algorithm, allowing us to sample from the target distribution, which is difficult to generate directly. The most traditional MCMC structural learning algorithm is the Structure MCMC algorithm proposed by [40], conducts MCMC sampling by adding, deleting, or reversing an edge in the network. Specifically, on a day *t*, let G and $G^{\prime }$ be two structures of a Bayesian network, where $G^{\prime }$ is obtained from G by adding, deleting, or reversing an edge. The Structure MCMC is a Metropolis-Hastings algorithm with an acceptance ratio, in our case,

$$\begin{array}{c} \alpha \left(G,G^{\prime }\right)=\text{min}\{1,\frac{P\left(G^{\prime }|D_{(t-w+1):t}\right)q\left(G|G^{\prime }\right)}{P\left(G|D_{(t-w+1):t}\right)q\left(G^{\prime }|G\right)}\}, \end{array}$$

where $P(G|D_{(t-w+1):t})$ is the BDeu score for the structure G using the data set *D*_(*t*−*w*+1):*t*_, and $q(G^{\prime }|G)$ is the transition kernel from the structure G to $G^{\prime }$. We take the structure with the highest BDeu score in the Structure MCMC as $G_{t}$.

Another algorithm we used in the study is the Order MCMC algorithm proposed by [41], which conducts MCMC sampling on the topological order space. The procedures of the Order MCMC algorithm are similar to those in the Structure MCMC algorithm, except we need to modify the BDeu score. We combine both algorithms to enhance the efficiency in terms of searching networks with high BDeu scores. The main advantage of using MCMC algorithms in our study is that it enables us to avoid becoming tapped in local maximum [51], making it more efficient to learn networks compared to some other optimization methods, such as greedy hill-climbing. This feature is especially important when it is necessary to learn many networks across thousands of trading days, as in our rolling-window modeling.

**Fig 3:**
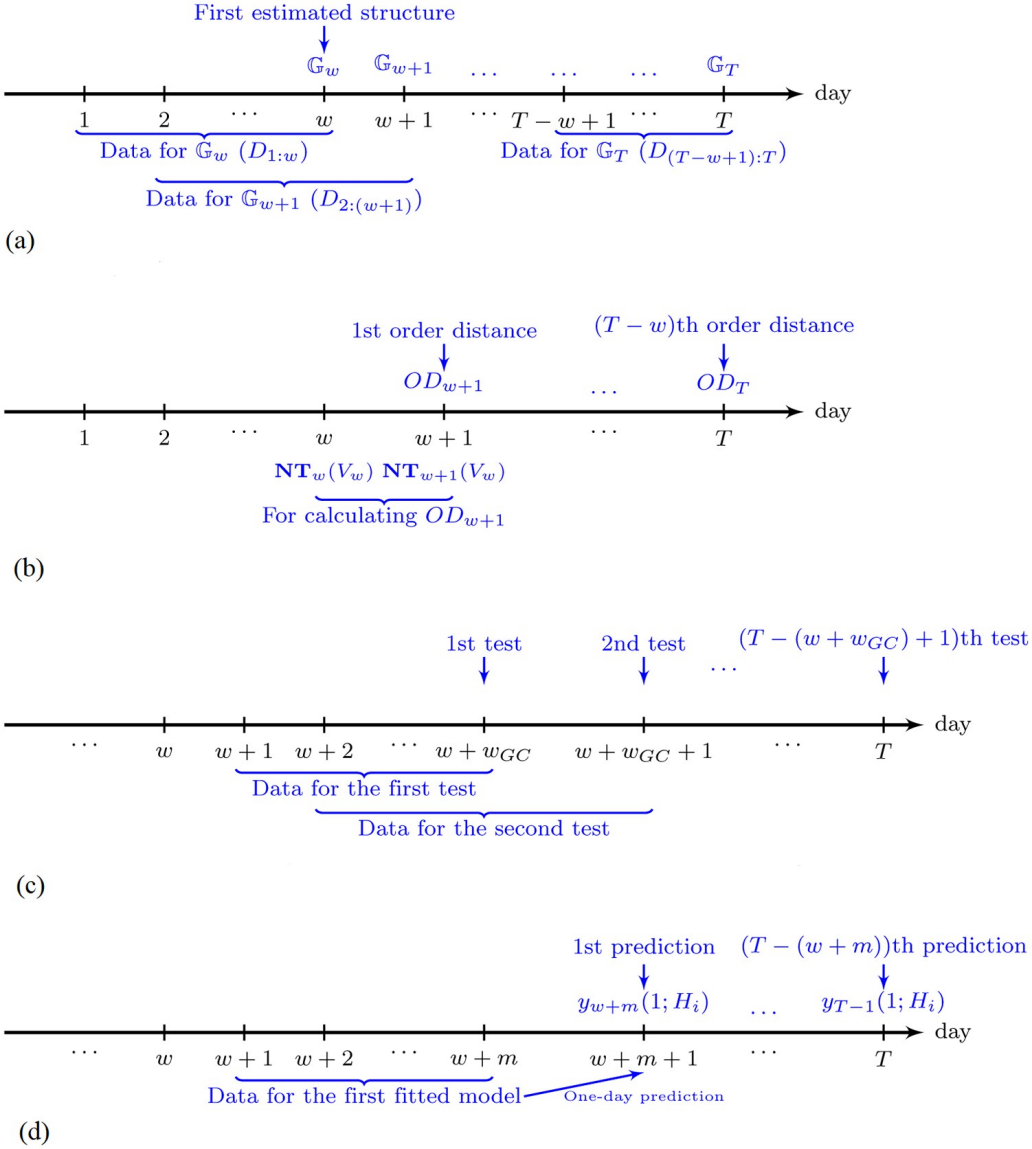
The timelines of the different steps in our study. (a) Timeline for the time series of network construction over the period under study, (b) Timeline for the calculation of the topological order distance over the period under study, (c) Timeline for the T − (w + wGC) + 1 Granger-causality tests over the period under study, and (d) Timeline for the LASSO regression prediction for the hypothesis H_i_ over the period under study, where i ∈ {0, 1*a*, 1*b*, 2}.

The maximum number of parents for each node, *M*, is set to 13. We choose *M* = 13 because the computation will be very slow if we were to use *M* > 13. Since we need *w* days of data to learn the structures, we can only obtain a time series of networks $\left\{G_{w},G_{w+1},\ldots ,G_{T}\right\}$. The workflow of the construction of the time series of networks is shown in the timeline in Fig 3a. The first estimated structure is on day *w* using the data set *D*_1:*w*_. The next structure to be estimated is on day *w* + 1 using the data set *D*_2:(*w*+1)_, and so on.

#### Granger-causality tests

**Fig 4:**
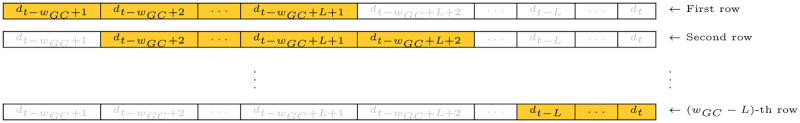
The data included when performing a Granger-causality test on day *t*, where *w*_*GC*_ = 40 and *L* = 1, 2, 3, 4, 5. *d*_*t*_ represents the data available on day *t*.

An objective of this paper is to apply the network statistics of Bayesian networks in financial risk management. We investigate if the modified network density in Eq (4) and the order distance in Eq (7) are helpful in predicting future losses and risks in the market. We use the HSI returns as a proxy of market losses. Let *R*_*t*_ be the return of HSI on day *t* and define the loss on day *t* by *Loss*_*t*_ = −min{*R*_*t*_, 0}. Following [17], we use a recent sample of observations to study any lead-lag relationships between historical network statistics and the loss on day *t*. In the empirical data analysis, we include observations from day *t* − *w*_*GC*_ + 1 to day *t* to conduct the Granger-causality tests by fitting the following regression models, where *w*_*GC*_ = 40 is the sample size:

$$\begin{array}{c} \left\{ \begin{array}{cc} H_{0}: & Loss_{t}=a_{0}+\sum_{i=1}^{L}a_{i}Loss_{t-i}+\varepsilon_{t}\\ H_{1a}: & Loss_{t}=a_{0}+\sum_{i=1}^{L}a_{i}Loss_{t-i}+\sum_{j=1}^{L}b_{j}MND\left(G_{t-j};M\right)+\varepsilon_{t}\\ H_{1b}: & Loss_{t}=a_{0}+\sum_{i=1}^{L}a_{i}Loss_{t-i}+\sum_{j=1}^{L}b_{j}OD_{t-j}+\varepsilon_{t}\\ H_{2}: & Loss_{t}=a_{0}+\sum_{i=1}^{L}a_{i}Loss_{t-i}+\sum_{j=1}^{L}b_{j}MND\left(G_{t-j};M\right)\\ & \;\;\;\;\;\;\;\;\;\;\;+\sum_{k=1}^{L}c_{k}OD_{t-k}+\sum_{\ell =1}^{L}d_{\ell }OD_{t-\ell }\times MND\left(G_{t-\ell };M\right)+\varepsilon_{t} \end{array},\right. \end{array}$$ (9)

where *a*_*i*_, *b*_*j*_, *c*_*k*_ and *d*_ℓ_ are regression coefficients, *ε*_*t*_’s are random errors in the regression models, and *L* is the number of lags included in the Granger-causality tests. The *H*_0_ represents the null model, which contains lagged losses as the only independent variables. The *H*_1*a*_ and *H*_1*b*_ respectively test whether the lagged modified network density and the order distance are helpful for the prediction of the next-day loss, and *H*_2_ includes both independent variables and their interaction terms. The Granger-causality tests in Eq (9) are conducted for *L* = 1, 2, 3, 4, 5. Note that, the sample size *w*_*GC*_ is always fixed. Fig 4 shows the use of data from day *t*−*w*_*GC*_ + 1 to *t* to perform one Granger-causality test. When we use a lag *L*, we use the first *L* observations as lagged values. Then, including the data on the current day, we use *L* + 1 days of values for each hypothesis in Eq (9). Thus, effectively, we have *w*_*GC*_−*L* rows of data to input into each Granger-causality test.

Similarly, we also conduct another set of Granger-causality tests in regard to the usefulness of the network statistics in predicting the volatility. A high level of volatility is associated with a high level of instability in financial markets and, thus, being able to predict future volatility movement can help predict market risk. The increase in volatility to a very high level can be associated with potential systemic risks in financial markets. A common proxy of volatility is the absolute return [44], and the corresponding null hypothesis is

$$\begin{array}{c} H_{0}:|R_{t}|=a_{0}+\sum_{i=1}^{L}a_{i}\left|R_{t-i}\right|+\varepsilon_{t}, \end{array}$$ (10)

with similar alternative hypotheses as in Eq (9), replacing *Loss*_*t*−*i*_ with |*R*_*t*−*i*_|, *i* = 0, 1, …, *L*. The workflow of the Granger-causality tests is summarized in Fig 3b and 3c. In Fig 3b, the first order distance that can be calculated is on day *w* + 1 since we need both **NT**_*w*_(*V*_*w*_) and **NT**_*w*+1_(*V*_*w*_) to calculate *OD*_*w*+1_ according to Eq (7), and the first available normalized topological order is **NT**_*w*_(*V*_*w*_) from the first network $G_{w}$. The next order distance *OD*_*w*+2_ is calculated from **NT**_*w*+1_(*V*_*w*+1_) and **NT**_*w*+2_(*V*_*w*+1_), and so on. Then, the first day on which we can conduct a Granger-causality test is on day *w* + *w*_*GC*_, using the risk indicators from day *w* + 1, the first day an order distance is available, to day *w* + *w*_*GC*_, leading to, altogether, *w*_*GC*_ days of observations, as shown in Fig 3c. The next test is conducted on day *w* + *w*_*GC*_ + 1, and so on. There are in total *T* − (*w* + *w*_*GC*_) + 1 days on which we conduct the Granger-causality tests.

#### LASSO regression prediction

**Fig 5:**
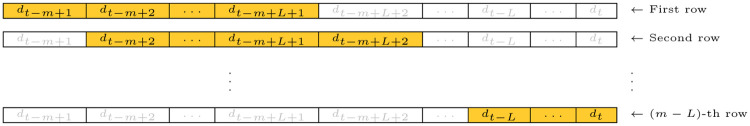
The data included when fitting a LASSO regression model on day *t*, where *m* = 40, *L* = 5 for models under *H*_1*a*_ and *H*_1*b*_, and *L* = 3 for models under *H*_2_. *d*_*t*_ represents the data available on day *t*.

In addition to the Granger-causality test, to investigate any significant leading effects of the modified network density and the order distance on the losses or absolute returns, we also evaluate the predictive performance using the models under four hypotheses in Eq (9) using the loss as the response, and that using the absolute return as response in Eq (10). A better prediction of volatility, as a trigger and a cause of financial crises, allows us to detect systemic risk events more accurately. On each day *t*, the most recent *m* = 40 days of observations (i.e., observations from day *t* − *m* + 1 to *t*) will be used to fit LASSO regression models for one-day predictions using the models under different hypotheses in Eq (9) and Eq (10). LASSO regressions may help reduce the variance and improve the prediction by shrinking some regression coefficients to zero [52], due to the penalty term. We will fix *L* = 5 for the models in *H*_1*a*_ and *H*_1*b*_ in the prediction but, unlike in Granger-causality tests, *L* = 1, 2, 3, 4 are not included because the LASSO regression will automatically shrink the coefficients to zero if some lags are irrelevant. For the models in *H*_2_, we pick a smaller number of lags *L* = 3, since this model contains more parameters than the models in *H*_1*a*_ and *H*_1*b*_. We need to set a smaller *L*, otherwise the degrees of freedom in the model will be too small, which may lead to overfitting. Similar to the mechanism within a Granger-causality test, we show the mechanism of fitting a LASSO regression model on a day *t* in Fig 5. The first *L* observations are used as lagged values. Including the observation on the current day, there are *L* + 1 days of values for each equation in Eq (9) and Eq (10). Thus, effectively, we have *m*−*L* rows of data to fit each LASSO regression model.

Let *y*_*t*_ be any one of the *Loss*_*t*_ or *R*_*t*_, as in the models in Eq (9) or Eq (10), respectively. We let *y*_*t*_(1;*H*_*i*_) be the one-day-ahead prediction of *y*_*t*+1_ using the model under the hypothesis *H*_*i*_, *i* ∈ {0, 1*a*, 1*b*, 2}, given the information up to time *t*. The root-mean-square error (RMSE) is used to evaluate the predictive performances

$$\begin{array}{c} RMSE\left(H_{i}\right)=\sqrt{\frac{1}{T-(w+m)}\sum_{t=w+m}^{T-1}{\left(y_{t}\left(1;H_{i}\right)-y_{t+1}\right)}^{2}}. \end{array}$$ (11)

The workflow of the regression prediction is summarized in Fig 3d. The first fitted model is on day *w* + *m*, using the data from day *w* + 1, the first day an order distance is available, to day *w* + *m*. We then conduct a one-day prediction for day *w* + *m* + 1. The next fitted model is on day *w* + *m* + 1, using the data from day *w* + 2 to *w* + *m* + 1. We then conduct a one-day prediction for day *w* + *m* + 2, and so on. The last prediction is on day *T*. Thus, there are a total of *T* − (*w* + *m*) predictions, as in the denominator of Eq (11).

On top of evaluating the overall performance, it is also meaningful to look into the predictive performance under tail scenarios (or extreme scenarios), which are more crucial to systemic risk. The following methodology (tail-event strategy) is specifically designed for developing a predictive strategy, where we believe that the network statistics based on the time series of Bayesian networks can be more effective in predicting market risk under tail scenarios. We propose using the predicted values of the loss or absolute return only when the observed loss or absolute return one day before the prediction is large, which may indicate the occurrence of tail scenarios in the next few days. In other words, we develop the following prediction scheme where we selectively use the predicted values generated by the network statistics to focus on tail scenarios, or to infer the systemic risk of the market. On each day *t*, we accept the prediction whenever the observed value, *y*_*t*_, is larger than an *m*′-day rolling-window *p*-th percentile *q*_*t*_(*p*) (i.e., the *p*-th percentile of {*y*_*t*_, *y*_*t*−1_, …, *y*_max{1,*t*−*m*′+1}_}). Otherwise, we do not include the predicted value in the RMSE assessment. Let *Q*(*p*) = {*t*:*y*_*t*_ > *q*_*t*_(*p*), *t* = *w* + *m*, …, *T*−1} be the set of time indexes when the predicted value *y*_*t*_(1, *H*_*i*_) is included in the RMSE assessment, as in (Eq (11)). We also use the RMSE to evaluate the predictive performance of the tail-event strategy. The RMSE only assesses the prediction on the days with large observed losses or absolute returns, the time indexes of which are in the set *Q*(*p*). The RMSE is then defined as

$$\begin{array}{c} RMSE_{p}\left(H_{i}\right)=\sqrt{\frac{1}{Q(p)}\sum_{t\in Q(p)}{\left(y_{t}\left(1;H_{i}\right)-y_{t+1}\right)}^{2}}. \end{array}$$ (12)

Both *RMSE*(*H*_*i*_) and *RMSE*_*p*_(*H*_*i*_) are used to assess the predictive performance of the modified network density and the order distance in regard to the loss and absolute return.

## Main results

### Visualization of financial networks

**Fig 6:**
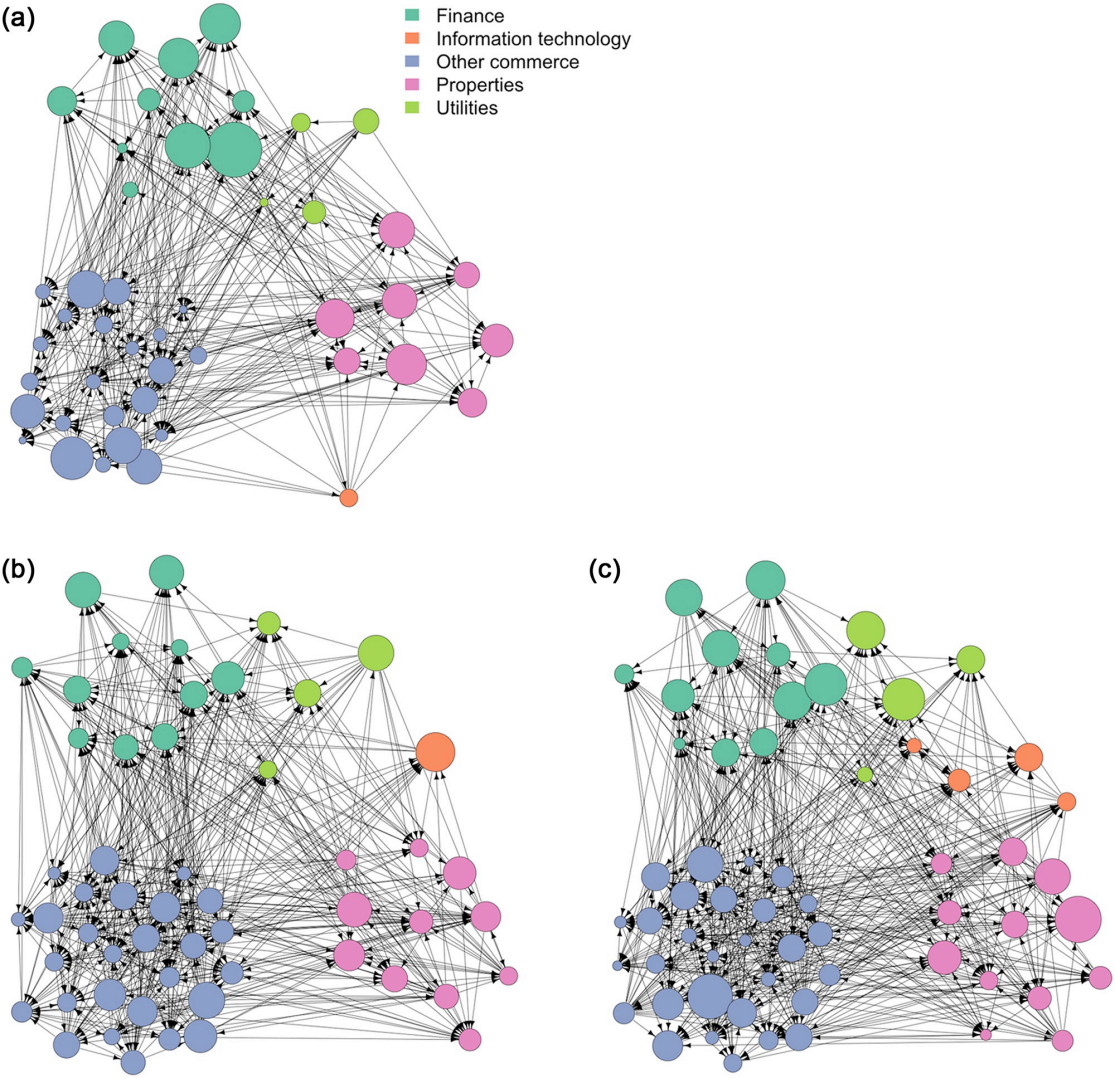
Visualization of the networks on three selected days. The black arrows represent edges and the circles represent nodes. The nodes are coloured by their sectors, and the sizes of the nodes are proportional to the 60-day moving average of the relative topological orders calculated using Eq (5). a) Visualization of the network on 27 October 2008 (during the global financial crisis. The HSI was at its lowest price on this day in 2008). (b) Visualization of the network on 26 January 2018. The HSI was at its highest price ever. c) Visualization of the network on 23 March 2020 (during the COVID-19). The HSI was at its lowest price on this day in 2020.

It is more efficient to explore the data across five sectors, classified according to the sub-indexes listed in [48]. The five sectors are: commerce (29 stocks), finance (11 stocks), information technology (four stocks), properties (12 stocks), and utilities (four stocks). The detailed description of these stocks are shown in the S1 Appendix in S1 File. The networks are learned using the window size *w* = 30 and the *T* = 3403 trading days of return indicators in Eq (8). The networks on three selected dates are shown in Fig 6. These are three example networks showing the importance of the order distance. Fig 6(a) shows the network on 27 October 2008 during the global financial crisis due to subprime mortgage problems [6]. Fig 6(b) shows the network on 26 January 2018, the day on which the HSI has the highest price ever. Fig 6(c) shows the network on 23 March 2020 during the COVID-19 pandemic. The HSI was at its lowest price of the year on this day in 2020. The connectedness of the networks is similar across sectors. The size of the nodes is proportional to the 60-day moving average of the relative topological order in Eq (5). The 60-day moving average is calculated to enable us to clearly visualize the trend of the relative topological order.

Fig 6 illustrates the behavior of the relative topological order on abnormal days (during the financial crisis of 2008 and the COVID-19 pandemic) and on a normal day. During the financial turmoil taking place on the days in Fig 6(a) and 6(c), the topological orders of the stocks are quite imbalanced; some nodes are very large while some are very small, showing that some stocks are more influential than the others. Fig 6(b) shows a network on 26 January 2018, within a period in which there was no particular extreme financial event. The 60-day moving average of the relative topological order of the stocks appears to be much more uniformly distributed, with similar node sizes. Fig 6 shows that the topological order of stocks is more stable during a normal period than in periods when high levels of financial uncertainty are anticipated. We believe that the relative topological order of stocks is associated with the stability of the market. Changes in the relative topological order can help identify adverse changes in the financial market. Therefore, we hypothesize that the topological order distance in Eq (7) can help track systemic risk when using Granger-causality tests and predictive analysis.

### Summary statistics of the financial networks

**Fig 7:**
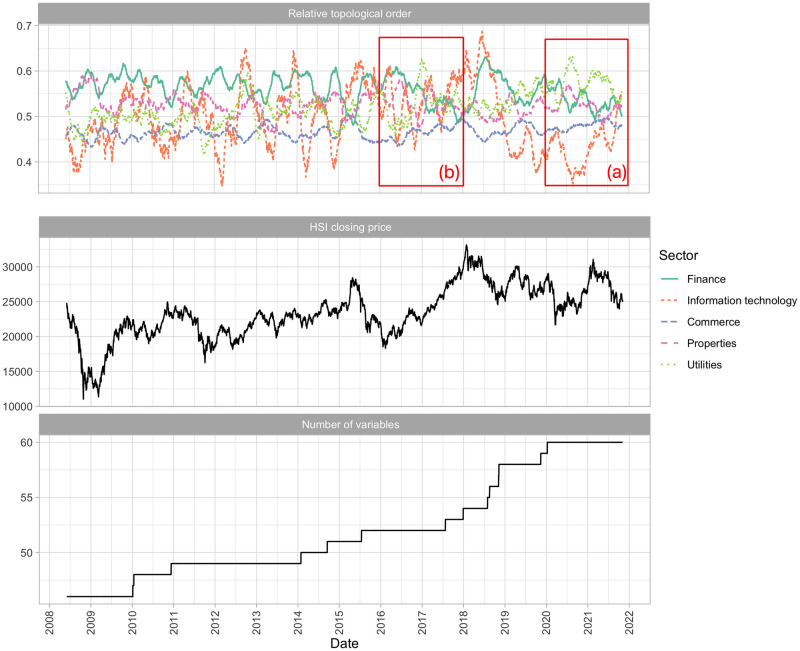
The time series plots of the smoothed average of the relative topological orders by sector, the daily closing price of the HSI and the number of variables in the networks from 2008 to 2021.

To summarize the networks in the period under study, Fig 7 shows the time series plot of the 60-day moving average of the relative topological order in Eq (5), with the stocks grouped by sector, the HSI closing prices, and the number of variables (or number of HSI’s constituent stocks) in the networks. In the first panel in Fig 7, the finance sector has a high relative topological order, except that the relative topological order of the utilities sector has increased above that of the finance sector during the COVID-19 pandemic. The topological order of the sectors changes over time, indicating that the market is led by different sectors at different periods. The topological orders change more rapidly in some periods; for example, during the COVID-19 pandemic in 2020, and in 2022, when the market was unstable (highlighted by the red rectangle marked with (a) in the first panel in Fig 7). The utilities sector is bumped up in the topological order, whereas the finance sector moves down. The lines representing the finance sector (green solid line) and the properties sector (pink dashed line) in the first panel in Fig 7 intersect several times during 2020 and 2022, indicating that the topological orders of these two sectors change rapidly. In contrast, the topological orders of the sectors from 2016 to 2018 (highlighted by the red rectangle marked with (b) in the first panel in Fig 7) are more stable, aside from the information technology sector, which contains only a few stocks. The finance sector (green solid line) has a high topological order during the period from 2016 to 2018, followed by the utilities sector (light green dotted line), the properties sector (pink dashed line), and the commerce sector (blue dashed line). Their topological orders stay at similar levels in this period, with only a few intersections. These examples show that the change in topological orders may be associated with the stability of the market. We quantify the degree of fluctuation of the relative topological order using the order distance in Eq (7), and conduct Granger-causality tests to predict and analyze the relationship between the topological orders and the systemic risk. The numbers of constituents in the HSI increases from 46 to 60, as shown in the fourth panel in Fig 7.

**Fig 8:**
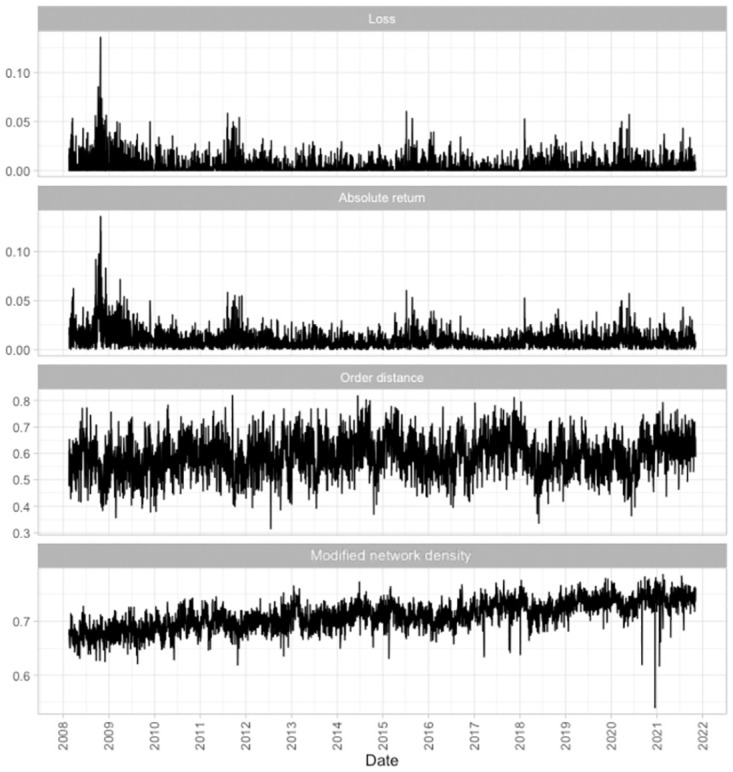
The time series of the responses (loss and absolute return) and the independent variables (order distance and modified network density) from 2008 to 2021.

Fig 8 shows the time series plots of the loss and absolute return of the HSI in the first two panels, and the order distance and modified network density of the networks in the third and fourth panels. Note that these statistics are not smoothed; they are directly used in the Granger-causality tests and rolling-window prediction study directly. In the first two panels, the loss and absolute return show some clustering patterns. We aim to predict the periods with extreme losses and absolute returns in order to establish early warning signals of possible systemic risk. In the third and fourth panels, the order distance and modified network density also show some clustering patterns, showing that the loss and absolute return may be associated with the order distance and the modified network density. Below, we evaluate the association, also the usefulness and performance of the order distance and modified network density in predicting the loss and absolute return, as two proxies of systemic risk below.

### Granger-causality tests

**Fig 9:**
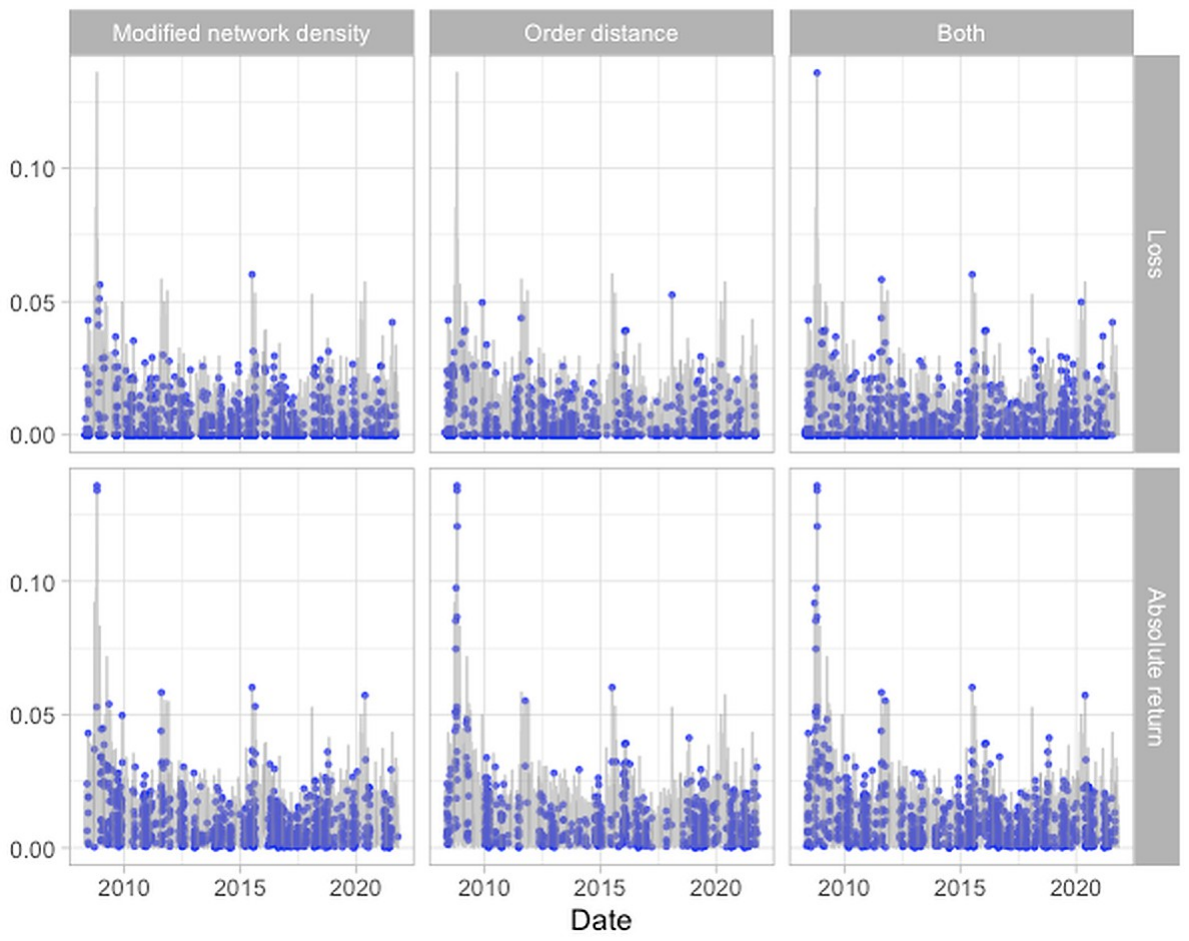
Time series plots of the loss and absolute return of the HSI, with blue dots indicating the data that are significant in the Granger-causality tests on the time points marked on the *x*-axis. There are six tests, the rows indicate the responses (loss and absolute return) and the columns represent the independent variables (Modified network density, order distance, and both of them) used in the Granger-causality tests.

The Granger-causality tests are conducted using the window size *w*_*GC*_ = 40, from day *w* + *w*_*GC*_ to day *T* (from 16 April 2008 to 04 November 2021), with a total of *T* − (*w* + *w*_*GC*_) + 1 = 3403 − 70 + 1 = 3334 trading days, as shown in the timeline in Fig 3c. Table 1 of day granger significant shows the numbers of trading days out of total 3,334 days that have at least one Granger-causality test, from lag 1 to lag 5, with significance at 0.1 level. The numbers range from 682 to 1,108, which correspond to around 20% to 33% of the trading days, showing that there is a high proportion of trading days on which the modified network density and the order distance “cause” the market loss or volatility in the Granger-causality sense. We find more significant results in regard to the loss than in regard to the absolute return. The results suggest that the order distance and modified network density are useful in predicting the absolute return and loss, and the evidence for this finding seems to be stronger when the target variable is the loss. Fig 9 shows the time series plots of the loss and absolute return, with blue dots denoting the trading days on which the loss and absolute return have significant results in the Granger-causality tests. The columns denote the variables used, from left to right: modified network density only (*H*_1*a*_), order distance only (*H*_1*b*_), and both (*H*_2_). There are more significant results detected after 2015. We can also observe from the figure that test results tend to be significant when there are big losses or high absolute returns. We explore the predictive power of extreme losses and extreme absolute returns below, using the RMSE in Eq (12).

**Table 1:** The number of trading days (out of total 3334 days) that have at least one Granger-causality test (from lag 1 to lag 5) with a significant result at 0.1 level. The rows are the responses and the columns are the hypotheses tested in the Granger causality tests stated in Eq (9) and Eq (10).

Target variables	*H*_1*a*_	*H*_1*b*_	*H*_2_
Loss	1108	890	1100
Absolute return	942	682	953

### Rolling-window prediction study

A LASSO regression prediction study is conducted with a sample size of *m* = 40, on each day from day *w* + *m* + 1 to day *T* (from 16 April 2008 to 03 November 2021), with a total of *T* − (*w* + *m*) = 3403 − (30 + 40) = 3333 days of predictions as shown in Fig 3d. The parameters in the LASSO regression models are tuned using time series cross-validation. The time series of the responses (loss and absolute return) and the independent variables (order distance and modified network density) are plotted in Fig 8. These variables are used to fit the two models in Eq (9) and Eq (10) under different hypotheses. One-day predictions are carried out for the different hypotheses of the two models on each day. The RMSEs of the prediction using a tail-event strategy with *m*′ = 100 are reported in Table 2. The first three parts labeled with “Extreme 0.1%”,“Extreme 1%”, and “Extreme 2%” correspond to the prediction under the tail scenarios, according to the strategy in Eq (12) by setting *p* = 99.9, 99, and 98, respectively. The last part labeled “all cases” shows the RMSEs calculated using Eq (11), if we conduct predictions for all trading days. The columns denote the models used according to the different hypotheses in Eq (9) and Eq (10). According to the tail-event strategy, the RMSEs of the absolute return are reduced by around 1.9% to 7% with different *p* in Eq (12) if we include only the order distance or include both the order distance and the network density as independent variables (the last two columns in Table 2, marked *H*_1*b*_ and *H*_2_). Note that, although using the modified network density alone (*H*_1*a*_) does not improve the prediction, including the order distance only (*H*_1*b*_) and including both the order distance and modified network density (*H*_2_) can both help to improve the predictive performance. Without using the tail-event strategy (the last part marked with “all cases” in Table 2), the models do not perform well. These results suggest that the order distance and modified network density are more useful for extreme cases. This phenomenon is consistent with the design of the tail-event strategy. Table 3 shows the number of days for which we actually accept the predictions with different *p* in Eq (12). There are 37, 69, and 116 days that we accept the predictions of the absolute returns for extreme 0.1%, 1%, and 2% scenarios respectively, which range from around 1% to 3.5% of the trading days out of the 3,333 days. Note that these numbers are the same across all *H*_1*a*_, *H*_1*b*_, and *H*_2_ for a fixed *p*, since the set *Q*(*p*) depends only on *p* and the observed data, but not on the predicted values.

**Table 2:** RMSEs of the prediction using models Eq (9) and Eq (10) respectively of different hypotheses when considering tail events (Extreme 0.1%, 1% and 2%) and all cases. The rows indicate the responses (loss and absolute return). Each RMSE of the predictive models in *H*_1*a*_, *H*_1*b*_ and *H*_2_ is compared with the RMSE of the predictive model in *H*_0_, using respectively the same data with the sets of time indexes *Q*(99.9), *Q*(99) and *Q*(98). The values inside the brackets are the percentage differences of the RMSE of the model in *H*_1*a*_, *H*_1*b*_ or *H*_2_ to the RMSE of the model in *H*_0_. A negative percentage means that the model under the alternative hypothesis has a smaller RMSE, i.e., the model under the alternative hypothesis performs better.

Case	Response/Model	*H*_0_	*H*_1*a*_	*H*_0_	*H*_1*b*_	*H*_0_	*H*_2_
Extreme	Loss	0.0392	0.0404 (3.2%)	0.0392	0.0387 (-1.1%)	0.0377	0.0375 (-0.5%)
0.1%	Absolute return	0.0392	0.0396 (1%)	0.0392	0.0365 (-7%)	0.0394	0.0372 (-5.6%)
Extreme	Loss	0.0349	0.0359 (2.8%)	0.0349	0.0349 (-0.1%)	0.0342	0.0340 (-0.6%)
1%	Absolute return	0.0338	0.0342 (1.2%)	0.0338	0.0320 (-5.4%)	0.0342	0.0326 (-4.6%)
Extreme	Loss	0.0310	0.0319 (2.7%)	0.0310	0.031 (-0.1%)	0.0306	0.0307 (0.3%)
2%	Absolute return	0.0305	0.0309 (1.4%)	0.0305	0.0298 (-2.2%)	0.0305	0.0299 (-1.9%)
All cases	Loss	0.0094	0.0098 (4.5%)	0.0094	0.0098 (4.8%)	0.0091	0.0096 (5.2%)
	Absolute return	0.0100	0.0106 (6%)	0.0100	0.0104 (4.9%)	0.0097	0.0104 (6.7%)

**Table 3:** The number of trading days out of 3333 days for which we accept the predictions in the RMSE assessments with extremes of 0.1%, 1%, and 2% in Eq (12). The rows indicate the responses (loss and absolute return) to predict.

	Response	Number of cases
Extreme 0.1%	Loss	43
	Absolute return	37
Extreme 1%	Loss	73
	Absolute return	69
Extreme 2%	Loss	105
	Absolute return	116

## Discussion

Volatility is can be considered as a trigger and a cause of a financial crisis, and it can therefore serve as a precautionary signal for systemic risks. The idea underlying the use of Bayesian networks to model the connectedness inside the financial market is that high levels of network connectedness can facilitate the transmission of market disturbance, thereby increasing the market volatility. By detecting the increase in network connectedness, we can be prepared before the market disturbance or changes spread, or the volatility increases substantially, signaling that high levels of systemic risks are evolving. An advantage of using Bayesian networks over undirected networks is that Bayesian networks allow stocks or financial assets to be ordered topologically.

We can see in Fig 6(a) and 6(c) that the relative topological orders of the stocks are quite imbalanced when there is financial turmoil. This can be explained by the cascading failures in highly connected financial networks. These failures are characterized by higher topological orders in a few nodes, such that any financial disturbance can flow to other stocks with lower topological orders, and eventually lead to the failure of the whole financial system. Although we find that the topological orders can be used to predict high levels of volatility, further research is needed in regard to the relationship between the change in topological orders and the flow of financial disturbance.

In Fig 9, we observe that the Granger-causality tests give significant results more often in the period from 2015 to 2021, a period with higher levels of network connectedness (as shown in the third panel in Fig 8), than in the period from 2008 to 2014. This phenomenon is consistent with the finding in [53] that higher levels of connectedness in the banking network within a country are associated with a higher probability of financial crises. More specifically, we can show that the two risk indicators (the modified network density and the order distances) are useful in detecting high levels of volatility, and also have good predictive performances for extreme cases. The Granger-causality tests using two indicators to detect high levels volatility yield significant results more often in the periods with higher levels of network connectedness, which are associated with a higher probability of financial crises. Moreover, our empirical study includes not only banks but also other financial sectors, showing that a similar phenomenon that occurs for banking networks, discussed in [53], also applies to a more general financial network.

The tail-event strategy used in the prediction study is specifically designed for predicting extreme events only. This prediction strategy is slightly atypical, but is useful in practice in this paper, in regard to the reduction in prediction RMSEs in Table 2. In practice, forecasting financial time series is a challenging task. A modest improvement in prediction would be beneficial for the practitioners [54]. The reduction in RMSEs in the predictions supports the idea that the modified network density and order distance can provide additional information to the predictive models. The use of modified network density and order distances is not limited to the LASSO prediction model we introduced. For example, we can include these two risk indicators into volatility models with regressors [55, 56].

Whereas the current results showed that the risk indicators help improve the predictability of the tail events, we still need to take into account the robustness of the methodology. We provide sensitivity analyses of *M* (the maximum number of parents for each node in the structural learning), *K* (the number of random samples used in the estimation of the median topological order), *w* (the number of days of data used in the structural learning), *m* (the number of days of data used to fit the LASSO regression prediction model), and *m*′ (the number of days of data included in the *m*′-day rolling-window *p*-th percentile) in the S4 to S7. Appendices in S1 File. Although the restriction on *M* is mainly due to the computational burden, it can affect the results. We show that the predictive performance is the best when taking *M* = 13 in the S4 Appendix in S1 File. We show that increasing the parameter *K* from 100 to a larger value can only provide a tiny benefit in terms of the accuracy of the estimation of the normalized topological order in the S5 Appendix in S1 File. Hence, the choice of *K* depends on the time available in practice. Even when we increase *K* from 100 to 1000, the RMSEs of the estimation of the normalized topological orders are only reduced from around 0.0017 to 0.0013, while the running time is increased by around 11 times, which is very inefficient. Moreover, the improvement is very small compared to the magnitude of the normalized topological order. The choices of the parameters *m* and *w*, however, are more restrictive; a longer window may include too much irrelevant information, while a shorter window will lead to unstable parameter estimation problems. We have shown that the choice of *w* = *m* = 30 is still good in terms of predictive performances (specifically, the prediction RMSEs show some improvements for the model under *H*_1*b*_ and *H*_2_ for the extreme 0.1% and 1%), while the choice *w* = *m* = 60 results in bad predictive performances in the S6 Appendix in S1 File, which show no improvement in all cases. The choice of *w* = 30 and *m* = 40 actually performs very well, as seen in the main result of this paper. In contrast, we show that the models perform well in terms of predictive RMSEs when the parameter *m*′ is respectively taken to be 40 and 60 in the S7 Appendix in S1 File (specifically, all the prediction RMSEs under the model *H*_1*b*_ and *H*_2_ are reduced for the extreme 0.1%, 1%, and 2%). The choice of *m*′ is then dependent on the expert knowledge of the person using the model, in regard to the horizon of interest. The physical meaning of *m*′ is that, we want to filter out an extreme day in a window of width *m*′. The proposed order distance indeed helps reduce the predictive performances in many different settings of the parameters. The sensitivity analysis shows a limitation of this study that the choice of parameters may affect the results. A possible way to enhance the robustness of the model is that instead of fixing the parameters, we can tune the parameters over time. One of the main purposes of this paper is to show that the order distances contain useful information for predicting volatility, and the LASSO prediction model is only one of the prediction models chosen to illustrate this. The order distances can be used as predictors in other models. Further research is needed to understand the behavior and predictive performance of the order distances in different models.

While we have shown that the methodology helps improve the predictive performance of the HSI, which is a representative index [49], we still need to test if the methodology works for other stock markets. We repeat the same modeling procedure using the Dow Jones Industrial Average Index in the S8 Appendix in S1 File. The closing prices of the DJIA Index and its constituent stocks from 19 September 2017 to 16 September 2022 (1,258 trading days) are collected. We conduct the same procedures in the study using the HSI in the main results. All settings for the parameters are also the same, except we use *M* = 7 and *M* = 13 for a sensitivity analysis on *M*. The RMSEs of the LASSO regression predictions using the equations in Eq (9) and Eq (10) with *M* = 7 and *M* = 13 are shown respectively in Table 11a and 11b S8 Appendix in the S1 File, and the RMSEs are reduced under models *H*_1*a*_ and *H*_2_ when we set *M* = 7, and the RMSEs are also reduced under model *H*_1*b*_ when we set *M* = 13. The methodology also improves the predictive performance for the DJIA Index in terms of prediction RMSEs. Hence, we believe that the methodology could be applied to different stock markets.

To conclude, we illustrate the usefulness of the order distance in terms of predicting the volatility using two sets of data: the HSI and DJIA Index. We provide a more detailed analysis on the HSI data in the Main Results section in this paper. The DJIA Index is also studied to demonstrate that the order distance also helps improve the prediction performance in another stock market. We show that there is a larger portion of days on which the lagged order distances and the modified network densities have significant Granger-causality in regard to the two proxies of the volatility, the absolute returns, and the loss, defined as the magnitude of the negative part of the returns. These two risk indicators, the order distance and the modified network density greatly improve the predictability of the absolute returns, which allows us to predict volatility more accurately, and thus prepare ourselves for possible systemic risk events. Note that, the order distance we proposed can only be measured using directed networks, which demonstrates the importance of the use of directed networks. The use of the order distance is not limited to the LASSO regression prediction model; we can include the order distance as a predictor in any volatility model to improve the predictability of tail events. In the future, we can continue to develop the application of the directed networks and the Bayesian networks in regard to the financial modeling and the systemic risk management, as the order distance is not the only information we can acquire from the Bayesian networks.

## Supporting information

S1 File (ZIP)
