# Supplementary material for: A moving-window bayesian network model for assessing systemic risk in financial markets
Source: PLoS One. 2023 Jan 20;18(1):e0279888. doi: 10.1371/journal.pone.0279888 (PMC9858016; doi:10.1371/journal.pone.0279888)
Supplement: S1 File — (ZIP) [file pone.0279888.s001.zip › supporting information.pdf]

Supporting Information

S1 Appendix: Details of the HSI constituent stocks

The names, stock symbols and sectors of the HSI constituent stocks, sorted by their sectors and stock symbols are shown in Table 4.

Constituent Name	Stock symbol	Sector
CKH HOLDINGS	0001.HK	Commerce
GALAXY ENT	0027.HK	Commerce
MTR CORPORATION	0066.HK	Commerce
GEELY AUTO	0175.HK	Commerce
ALI HEALTH	0241.HK	Commerce
CITIC	0267.HK	Commerce
WH GROUP	0288.HK	Commerce
SINOPEC CORP	0386.HK	Commerce
TECHTRONIC IND	0669.HK	Commerce
CHINA UNICOM	0762.HK	Commerce
PETROCHINA	0857.HK	Commerce
XINYI GLASS	0868.HK	Commerce
CNOOC	0883.HK	Commerce
CHINA MOBILE	0941.HK	Commerce
XINYI SOLAR	0968.HK	Commerce
HENGAN INT'L	1044.HK	Commerce
CSPC PHARMA	1093.HK	Commerce
SINO BIOPHARM	1177.HK	Commerce
BYD COMPANY	1211.HK	Commerce
BUD APAC	1876.HK	Commerce
SANDS CHINA LTD	1928.HK	Commerce
AAC TECH	2018.HK	Commerce
ANTA SPORTS	2020.HK	Commerce
WUXI BIO	2269.HK	Commerce
SHENZHOU INTL	2313.HK	Commerce
MENGNIU DAIRY	2319.HK	Commerce
LI NING	2331.HK	Commerce
SUNNY OPTICAL	2382.HK	Commerce
HAIDILAO	6862.HK	Commerce
HSBC HOLDINGS	0005.HK	Finance
HANG SENG BANK	0011.HK	Finance
HKEX	0388.HK	Finance
CCB	0939.HK	Finance
AIA	1299.HK	Finance
ICBC	1398.HK	Finance
PING AN	2318.HK	Finance
BOC HONG KONG	2388.HK	Finance
CHINA LIFE	2628.HK	Finance
CM BANK	3968.HK	Finance
BANK OF CHINA	3988.HK	Finance
TENCENT	0700.HK	Information technology
XIAOMI-W	1810.HK	Information technology
MEITUAN-W	3690.HK	Information technology
BABA-SW	9988.HK	Information technology
HENDERSON LAND	0012.HK	Properties
SHK PPT	0016.HK	Properties

NEW WORLD DEV	0017.HK	Properties
HANG LUNG PPT	0101.HK	Properties
CHINA OVERSEAS	0688.HK	Properties
LINK REIT	0823.HK	Properties
LONGFOR GROUP	0960.HK	Properties
CHINA RES LAND	1109.HK	Properties
CK ASSET	1113.HK	Properties
WHARF REIC	1997.HK	Properties
COUNTRY GARDEN	2007.HK	Properties
CG SERVICES	6098.HK	Properties
CLP HOLDINGS	0002.HK	Utilities
HK & CHINA GAS	0003.HK	Utilities
POWER ASSETS	0006.HK	Utilities
CKI HOLDINGS	1038.HK	Utilities

Table 4: The names, stock symbols and sectors of the HSI constituent stocks, sorted by their sectors and stock symbols.

S2 Appendix: Proof of the formula of the maximum number of possible number of edges in a Bayesian network given a restriction of parent sizes

To calculate such maximum number, consider the DAG in Figure 10 which has a network of $n = 5$ nodes and we restrict the maximum number of arcs of each node to be $M = 3$. Three arcs from X_4, X_3 and X_2 are first connected to X_1 (colored in blue). Three arcs from X_5, X_4 and X_3 are then connected to X_2 (colored in red). Two arcs from X_5, X_4 are then connected to X_3 (colored in light green). Finally, an arc from X_5 is connected to X_4 (colored in brown). The numbers of parents of the nodes X_1 to X_5 are respectively $3, 3, 3, 2$, and 1. The only topological order of this network is $(X_5, X_4, X_3, X_2, X_1)$, such that X_4 can at most have one parent, which is X_5, and X_5 cannot have any parents. All nodes X_i with $i \leq 3$ contain the maximum of $M = 3$ parents. Thus, the maximum possible number of arcs of a network of $n = 5$ nodes with a restriction on the maximum number of parents of each node $M = 3$ is equal to $3 + 3 + 2 + 1 = 9$. In general, for a network with n nodes, we can construct a network by connecting $X_{i+1}, \ldots, X_{\min\{i+M,n\}}$ to X_i for $i = 1, \ldots, n-1$, and the maximum possible number of arcs of a network of $\sum_{i=1}^{n-1} \min\{M, i\} = nM - M(M+1)/2$. Consider the DAG in Figure 10 which has a network of $n = 5$ nodes and we restrict the maximum number of arcs of each node to be $M = 3$. Three arcs from X_4, X_3 and X_2 are first connected to X_1 (colored in blue). Three arcs from X_5, X_4 and X_3 are then connected to X_2 (colored in red). Two arcs from X_5, X_4 are then connected to X_3 (colored in light green). Finally, an arc from X_5 is connected to X_4 (colored in brown). The numbers of parents of the nodes X_1 to X_5 are respectively $3, 3, 3, 2$, and 1. The only topological order of this network is $(X_5, X_4, X_3, X_2, X_1)$, such that X_4 can at most have one parent, which is X_5, and X_5 cannot have any parents. All nodes X_i with $i \leq 3$ contain the maximum of $M = 3$ parents. Thus, the maximum possible number of arcs of a network of $n = 5$ nodes with a restriction on the maximum number of parents of each node $M = 3$ is equal to $3 + 3 + 2 + 1 = 9$. In general, for a network with n nodes, we can construct a network by connecting $X_{i+1}, \ldots, X_{\min\{i+M,n\}}$ to X_i for $i = 1, \ldots, n-1$, and the maximum possible number of arcs of a network of $\sum_{i=1}^{n-1} \min\{M, i\} = nM - M(M+1)/2$.

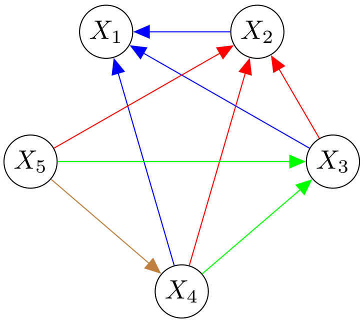

Figure 10: Illustration of the construction a network with the maximum number of arcs with number of nodes $n = 5$ and the maximum number of parents of each node $M = 3$. Three arcs from X_4, X_3 and X_2 are first connected to X_1 (colored in blue). Three arcs from X_5, X_4 and X_3 are then connected to X_2 (colored in red). Two arcs from X_5, X_4 are then connected to X_3 (colored in light green). Finally, an arc from X_5 is connected to X_4 (colored in brown).

S3 Appendix: Empirical evidence on the claim that a higher level of volatility may be associated with a more rapid change in the network

We provide empirical evidence that the association between the volatility and order distance is more likely to be positive whenever the volatility is high. Note that the association also changes in time, since we are using a rolling-window approach, such that we observe that the association between the volatility and the order distance is sometimes negative and sometimes positive. To support the above claim, we want to check how often the association between the volatility and the order distance is positive, at different levels of volatility. This can be done by fitting a rolling-window regression to the lagged order distances on the absolute returns, a proxy of the volatility. We will compare the proportions of the slopes of the lagged order distances that are positive at different levels of absolute returns. If the proportions of positive slopes are higher when the levels of absolute returns are larger, then the above claim is supported.

Two sets of data are used to support this claim: The daily returns of the Hang Seng Index and its constituent stocks from 2 January 2008 to 4 November 2021 ($T = 3,403$ trading days in this case), and the daily returns of the Dow Jones Industrial Average Index and its constituent stocks from 19 September 2017 to 16 September 2022 ($T = 1,257$ trading days in this case). For each data set, the Bayesian networks for each of the trading days are learned using the return indicators in Eq. (8) in the paper using a rolling-window size of $w = 30$, and the order distances between all consecutive trading days are calculated. Recall that we denote the network on day t as $\mathbb{G}_t$, the order distance between $\mathbb{G}_{t-1}$ and $\mathbb{G}_t$ as OD_t, and the modified network density on $\mathbb{G}_t$ as $MND(\mathbb{G}_t; M)$. To investigate the association between the volatility and how rapidly the network changes (measured using the order distance), we fit the regression

$$|R_t| = \beta_{0t} + \beta_{1t} \times OD_{t-1} + \beta_{2t} \times MND(\mathbb{G}_{t-1}; M) + \beta_{3t} \times |R_{t-1}| + \varepsilon_i, \tag{13}$$

using the data from day $t - m + 1$ to day t (a $m = 40$-day rolling-window), where ε_i is the regression error term. The absolute return $|R_t|$ is used as a proxy of the volatility. This regression model is similar to those in Eq. (9) and Eq. (10) in the paper, which are used for the Granger-causality tests and the LASSO regression prediction study in this paper, but only include the first lagged values. We want to investigate the association between the volatility and the order distance (i.e., the sign of β_{1t}). Since the order distances are autocorrelated (as shown in Figure 11a for the HSI data, and in Figure 11b for the DJIA Index data, where the HSI data shows a high persistent pattern, while the DJIA Index is less persistent but still feature a few significant lags), it is sufficient to include the first lagged order distance in Eq. (13) for the purpose of investigating the association. The terms $MND(\mathbb{G}_{t-1}; M)$ and $|R_{t-1}|$ are used as control variables in the regression.

We fit the regression in Eq. (13) for trading days $t = w+m, \dots, T$, and obtained the estimates of β_{1t}'s, symbolically, $\{b_{w+m}, b_{w+m+1}, \dots, b_T\}$. To quantify that how likely a higher level of volatility is positively associated with the rapidness of the network changes (measured using order distance), we first partition the trading days by their log-transformed absolute returns (log-transformation is conducted to enable us to better visualize the data) $\log |R_t|$: $(-\infty, -8], (-8, -7.6], (-7.6, -7.2], \dots, (-3.6, -3.2], (-3.2, \infty)$. The intervals are selected such that each interval contains sufficient. Then, for each interval, we calculate the proportion of b_t that are positive. The line in Figure 12a shows the proportions of b_t that are positive in each of the the above intervals for the HSI data, and the histogram shows the number of trading days in each of the intervals. The line shows an increasing trend, as the log absolute return increases except in the last interval, which contains only a few trading days, indicating that the absolute returns (the proxy of the volatility) are more often positively associated with the order distances when the absolute returns are large (i.e., when the market is volatile). Figure 12b shows the similar results for the DJIA Index data. The increasing trend is even more obvious than the case for the HSI.

The implication is that, the order distance is useful for predicting volatility in the periods with high levels volatility, and a high level of volatility is often accompanied by a large order distance. Note that a "large" order distance here means it is relatively large compared to the recent trading days. Moreover, the rolling-window regression only considers the most recent 40 days of data. It is not appropriate, for example, to compare the order distances today and two years ago, since seasonal patterns are observed in the time series plot of the order distance in the third panel in Figure 8 in the paper, indicating that the overall levels of order distances in different windows could be different.

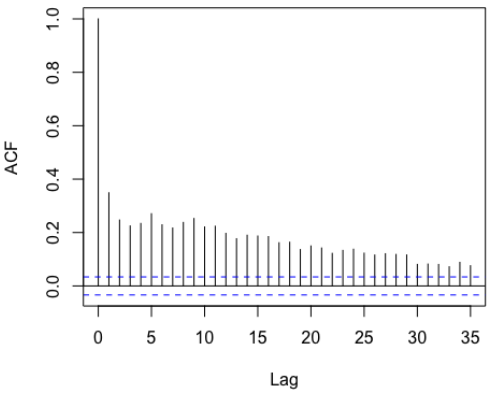

(a) The ACF of OD_t of the HSI data.

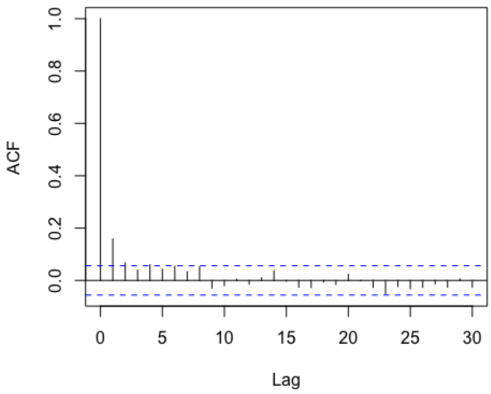

(b) The ACF of OD_t of the DJIA Index data.

Figure 11: The autocorrelation functions (ACF) of OD_t's in the HSI data and the DJIA Index data.

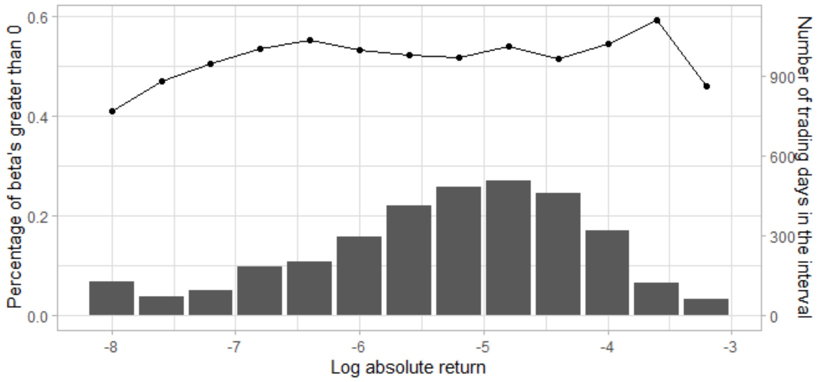

(a) HSI data.

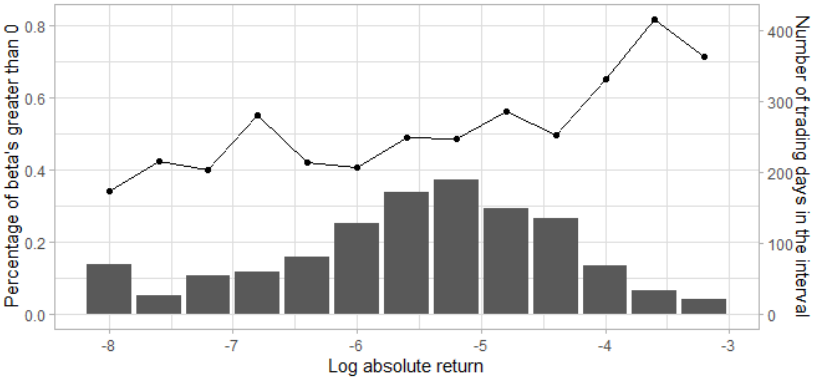

(b) DJIA Index data.

Figure 12: Each plot shows the proportions of b_t that are positive in the intervals $(-\infty,-8],(-8,-7.6],\ldots,(-3.6,-3.2],(-3.2,\infty)$. The dots represent the upper bounds of the intervals on the horizontal axis, and the percentage of b_t that are positive on the left vertical axis. The histogram below the line plot shows the numbers of trading days in each of the intervals, as marked on the right vertical axis.

S4 Appendix: Sensitivity analysis for the parameter M, the maximum number of parents for each node in the structural learning

To test the effect of the choice of M, we repeat the LASSO rolling-window prediction study using $M = 4, 6, 9, 11$ for the HSI data. The settings of the other parameters remain unchanged. The prediction RMSEs of the prediction using different M are also reported in Table 5. The performances are good for $M = 4$ and $M = 11$ while we do not observe any improvement for $M = 6$ and $M = 9$. The result using $M = 13$ in Table 2 is the best in terms of percentage reduction compared to these cases. However, it is also meaningful to compare the values of the RMSEs directly, since, the tail-event strategy only depends on the choice of m', the number of days of data included in the m'-day rolling window p-th percentile, which means that, fixing m', the samples included in the RMSE assessment are the same regardless of which M we use. The values of RMSEs under H_{1b} and H_2 using $M = 13$, shown in Table 2, are almost all smaller than those using other M's. The performance using $M = 13$ is overall the best not only in terms of percentage reduction, but also the values of RMSEs themselves. A larger M performs better. However, we do not provide results for $M > 13$ due to the computational burden involved. We cannot conclude that a larger M improves the predictive performance; instead, we show that the order distances are useful for some M, and thus, in practice, we may need to tune the parameter M carefully.

Although the restriction on M is mainly due to the computational burden, it can affect the results. Alternatively, we can also tune the parameter M using time series cross-validation, but then, the computational time is expected to be much longer, since we would need to repeatedly learn the networks using different values of M, and check the prediction performances using these learned networks. Hence, we use $M = 13$ because we do not want to impose too many restrictions on the structural learning. Equally, we do not want to computational time to be too long. Table 6 provides the total running time for learning all networks in the HSI data on $T - w + 1 = 3403 - 30 + 1 = 3374$ trading days. It takes around 3.4 days to finish the learning for $M = 11$. However, when we increase M from 11 to 13, it takes an additional 1.74 days of running time. We expect the running time required to be at an order that is larger than $O(M)$. Then, $M = 13$ is a fair choice to balance the running time while allowing the networks to be sufficiently flexible.

Case	Response/Model	H_0	H_{1a}	H_0	H_{1b}	H_0	H_2
Extreme	Loss	0.0392	0.0380(**-3.0%**)	0.0392	0.0407(3.7%)	0.0377	0.0379(0.7%)
0.1%	Absolute return	0.0392	0.0391(**-0.1%**)	0.0392	0.0395(0.7%)	0.0394	0.0384(**-2.5%**)
Extreme	Loss	0.0349	0.0341(**-2.3%**)	0.0349	0.0358(2.5%)	0.0342	0.0343(0.3%)
1%	Absolute return	0.0338	0.0340(0.6%)	0.0338	0.0344(2.0%)	0.0342	0.0336(**-1.7%**)
Extreme	Loss	0.0310	0.0305(**-1.8%**)	0.0310	0.0318(2.2%)	0.0306	0.0305(**-0.2%**)
2%	Absolute return	0.0305	0.0306(0.3%)	0.0305	0.0312(2.5%)	0.0305	0.0301(**-1.3%**)
All cases	Loss	0.0094	0.0096(3.1%)	0.0094	0.0097(4.1%)	0.0091	0.0098(7.5%)
	Absolute return	0.0100	0.0104(3.7%)	0.0100	0.0105(6.0%)	0.0097	0.0102(5.3%)

(a) $M = 4$

Case	Response/Model	H_0	H_{1a}	H_0	H_{1b}	H_0	H_2
Extreme	Loss	0.0392	0.0414(5.5%)	0.0392	0.0396(1.0%)	0.0377	0.0406(7.6%)
0.1%	Absolute return	0.0392	0.0397(1.5%)	0.0392	0.0397(1.5%)	0.0394	0.0400(1.5%)
Extreme	Loss	0.0349	0.0365(4.5%)	0.0349	0.0351(0.5%)	0.0342	0.0360(5.2%)
1%	Absolute return	0.0338	0.0344(2.0%)	0.0338	0.0344(2.0%)	0.0342	0.0347(1.5%)
Extreme	Loss	0.0310	0.0329(6.0%)	0.0310	0.0312(0.5%)	0.0306	0.0350(14.4%)
2%	Absolute return	0.0305	0.0309(1.5%)	0.0305	0.0308(1.0%)	0.0305	0.0310(1.7%)
All cases	Loss	0.0094	0.0120(27.9%)	0.0094	0.0096(3.1%)	0.0091	0.0108(18.8%)
	Absolute return	0.0100	0.0150(50.8%)	0.0100	0.0103(3.8%)	0.0097	0.0118(21.3%)

(b) $M = 6$

Case	Response/Model	H_0	H_{1a}	H_0	H_{1b}	H_0	H_2
Extreme	Loss	0.0392	0.0401(2.4%)	0.0392	0.0404(2.9%)	0.0377	0.0383(1.7%)
0.1%	Absolute return	0.0392	0.0400(2.0%)	0.0392	0.0401(2.5%)	0.0394	0.0405(2.8%)
Extreme	Loss	0.0349	0.0355(1.7%)	0.0349	0.0356(1.9%)	0.0342	0.0349(2.0%)
1%	Absolute return	0.0338	0.0342(1.4%)	0.0338	0.0345(2.2%)	0.0342	0.0344(0.5%)
Extreme	Loss	0.0310	0.0315(1.4%)	0.0310	0.0317(2.1%)	0.0306	0.0311(1.7%)
2%	Absolute return	0.0305	0.0309(1.3%)	0.0305	0.0309(1.4%)	0.0305	0.0305(**-0.1%**)
All cases	Loss	0.0094	0.0122(30.0%)	0.0094	0.0099(5.7%)	0.0091	0.0099(8.7%)
	Absolute return	0.0100	0.0121(21.3%)	0.0100	0.0104(4.6%)	0.0097	0.0117(20.3%)

(c) $M = 9$

Case	Response/Model	H_0	H_{1a}	H_0	H_{1b}	H_0	H_2
Extreme	Loss	0.0392	0.0405(3.3%)	0.0392	0.0395(0.8%)	0.0377	0.0394(4.5%)
0.1%	Absolute return	0.0392	0.0407(3.9%)	0.0392	0.0380(**-2.9%**)	0.0394	0.0384(**-2.7%**)
Extreme	Loss	0.0349	0.0360(3.0%)	0.0349	0.0348(**-0.3%**)	0.0342	0.0350(2.2%)
1%	Absolute return	0.0338	0.0350(3.6%)	0.0338	0.0334(**-1.1%**)	0.0342	0.0338(**-1.1%**)
Extreme	Loss	0.0310	0.0320(3.0%)	0.0310	0.0311(0.0%)	0.0306	0.0313(2.2%)
2%	Absolute return	0.0305	0.0310(1.8%)	0.0305	0.0302(**-1.0%**)	0.0305	0.0299(**-1.9%**)
All cases	Loss	0.0094	0.0097(3.8%)	0.0094	0.0097(3.9%)	0.0091	0.0096(5.0%)
	Absolute return	0.0100	0.0104(4.2%)	0.0100	0.0103(3.5%)	0.0097	0.0102(5.2%)

(d) $M = 11$

Table 5: The RMSEs of the LASSO rolling-window prediction study using different values of M.

M	Time (in days)
4	2.65
6	2.51
9	2.70
11	3.40
13	5.14

Table 6: The average times (in days) taken to complete the structural learning.

S5 Appendix: Sensitivity analysis for the parameter K, the number of random samples used in the estimation of the median topological order

We use $K = 100$ for the main results for the estimation of $M_t(X_i)$, the median of all possible topological orders $T_t(X_i)$. We use $M_t(X_i)$ to calculate $\mathbf{NT}_t(V_s)$, the vector of normalized topological orders on day t with respect to the set V_s. It is therefore important to evaluate the effects when K is altered, since $\mathbf{NT}_t(V_s)$ is used as a predictor in our study, which is one of the contributions of the paper. Let $\mathbf{NT}_t^k(V_t)$ be the estimator of the true vector of normalized topological orders when we take $K = k$ in the estimation of $M_t(X_i)$. We aim to evaluate, at different $K = k$, the RMSE of $\mathbf{NT}_t^k(V_t)$:

$$RMSE(\mathbf{NT}_t^k(V_t)) = \sqrt{E\left[(\mathbf{NT}_t^k(V_t) - E(\mathbf{NT}_t^k(V_t)))^T(\mathbf{NT}_t^k(V_t) - E(\mathbf{NT}_t^k(V_t)))\right]} \tag{14}$$

Eq. (14) is the square root of the sum of squared errors of all components in the vector $\mathbf{NT}_t^k(V_t)$, which can be interpreted as the total error of the vector. However, the close form of Eq. (14) is unknown. We conduct a simulation and generate $n_{rmse} = 100$ observations of $\mathbf{NT}_t^k(V_t)$, namely $([\mathbf{NT}_t^k(V_t)]_1, [\mathbf{NT}_t^k(V_t)]_2, \ldots, [\mathbf{NT}_t^k(V_t)]_{n_{rmse}})$, and estimate Eq. (14) using

$$\widehat{RMSE}(\mathbf{NT}_t^k(V_t)) = \sqrt{\frac{1}{n_{rmse}} \sum_{i=1}^{n_{rmse}} ([\mathbf{NT}_t^k(V_t)]_i - \overline{\mathbf{NT}_t^k(V_t)})^T([\mathbf{NT}_t^k(V_t)]_i - \overline{\mathbf{NT}_t^k(V_t)})}, \tag{15}$$

where $\overline{\mathbf{NT}_t^k(V_t)} = \frac{1}{n_{rmse}} \sum_{i=1}^{n_{rmse}} \left[\mathbf{NT}_t^k(V_t)\right]_i$.

We calculate Eq. (15) for $t = w, w+1, \ldots, T$ and $K = 10, 20, \ldots, 90, 100, 200, 300, 500, 1000$. The estimated RMSEs are illustrated in Figure 13; the horizontal axis indicates different values of K, and the vertical axis indicates the values of the estimated RMSEs. Each boxplot shows the distribution of the estimated RMSE for $t = w, w + 1, \ldots, T$ at the specified K. The RMSEs, which account for the total errors, are ranged from around 0.001 to 0.0025 when $K = 100$. Since the number of stocks included in the study ranges from 46 to 60, a normalized topological order of a node would have a magnitude around $1/46 \approx 0.022$ to $1/60 \approx 0.017$ (since these normalized topological orders sum to 1), which is much larger than the RMSEs. Hence, using $K = 100$ is sufficient, since the errors are much smaller than the magnitude of a normalized topological order. The average time (in minutes) to sample K topological orders for estimating a median topological order are shown in Table 7. The average time taken for sampling $K = 100$ topological orders is 0.0358 seconds. Increasing K can actually increase the accuracy of estimating the normalized topological orders, but the improvement is not proportional to the cost of time. For example, if we alter K from 100 to 1000, the RMSEs can be reduced from around 0.0017 to 0.0013, as shown in Figure 13. However, the computational times to estimate a normalized topological orders increases from 0.0358 seconds to 0.3923 seconds, which is around 11 times longer. It took around two hours to estimate all of the order distances using $K = 100$. Hence, we choose $K = 100$, since taking a larger K can only provide a tiny benefit in terms of the accuracy of the estimation. Alternatively, if time allows, we can choose to use a larger K in practice.

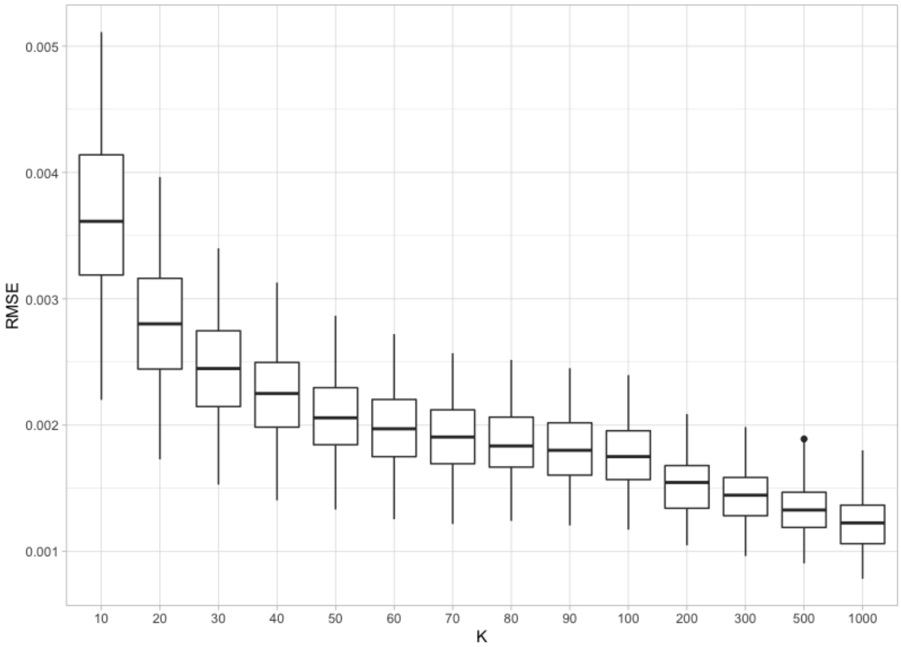

Figure 13: A time series of boxplots of the estimated RMSE in Eq. (15). The horizontal axis indicates different values of K, and the vertical axis indicates the values of the estimated RMSEs. Each boxplot shows the distribution of the estimated RMSE for $t = w, w+1, \ldots, T$ at the specified K.

K	Time (in seconds)
10	0.0038
20	0.0062
30	0.0094
40	0.0136
50	0.0165
60	0.0197
70	0.0230
80	0.0295
90	0.0300
100	0.0358
200	0.0759
300	0.1021
500	0.1765
1000	0.3923

Table 7: The average times (in seconds) taken to sample $K = 10, 20, \ldots, 90, 100, 200, 300, 500, 1000$ topological orders in order to estimate a median topological order.

S6 Appendix: Sensitivity analysis for the parameter w, the number of days of data used in the structural learning, and m, the number of days of data used to fit the LASSO regression prediction model

We set $w = 30$ and $m = 40$ for the models in the main results. Note that the choices of the parameters w and m are more restrictive than the other parameters. A longer window may include too much irrelevant information, while a shorter window will lead to unstable parameter estimation problems. Table 8a and Table 8b show the RMSEs of the LASSO prediction with $w = m = 30$ and $w = m = 60$, respectively. We can still observe improvements in predictive performances when we set $w = m = 30$, whereas the prediction becomes worse if we set $w = m = 60$. Note that, although the percentages RMSE reduced in Table 8a (ranging from 1.2% to 3.2%) are not as good as those in Table 2 (ranged from -0.1% to 7%), the values of the RMSEs under H_{1b} and H_2 in Table 8a and Table 2 are actually very close. Moreover, the tail-event strategy only depends on the choice of m', the number of days of data included in the m'-day rolling window p-th percentile; hence, the samples included in the RMSE assessment are the same. Then, setting $w = m = 30$ actually performs similarly well under H_{1b} and H_2. Both cases $w = m = 30$, and $w = 30, m = 40$ also support the notion that the order distances help improve the predictive performance.

Case	Response/Model	H_0	H_{1a}	H_0	H_{1b}	H_0	H_2
Extreme	Loss	0.0375	0.0388(3.5%)	0.0375	0.0370(**-1.2%**)	0.0375	0.0376(0.4%)
0.1%	Absolute return	0.0387	0.0404(4.5%)	0.0387	0.0375(**-3.2%**)	0.0387	0.0381(**-1.5%**)
Extreme	Loss	0.0345	0.0351(1.8%)	0.0345	0.0348(0.9%)	0.0345	0.0349(1.3%)
1%	Absolute return	0.0336	0.0344(2.4%)	0.0336	0.0328(**-2.4%**)	0.0336	0.0328(**-2.4%**)
Extreme	Loss	0.0308	0.0314(1.9%)	0.0308	0.0311(1.0%)	0.0308	0.0313(1.5%)
2%	Absolute return	0.0301	0.0310(3.0%)	0.0301	0.0303(0.6%)	0.0301	0.0309(2.7%)
All cases	Loss	0.0092	0.0095(3.5%)	0.0092	0.0096(4.4%)	0.0092	0.0099(6.9%)
	Absolute return	0.0098	0.0103(5.0%)	0.0098	0.0102(3.4%)	0.0098	0.0106(7.9%)

(a) $w = m = 30$

Case	Response/Model	H_0	H_{1a}	H_0	H_{1b}	H_0	H_2
Extreme	Loss	0.0390	0.0393(0.6%)	0.0390	0.0393(0.8%)	0.0386	0.0388(0.5%)
0.1%	Absolute return	0.0400	0.0400(0.1%)	0.0400	0.0403(0.7%)	0.0395	0.0397(0.6%)
Extreme	Loss	0.0305	0.0353(0.7%)	0.0350	0.0353(0.9%)	0.0352	0.0356(1.1%)
1%	Absolute return	0.0342	0.0345(0.7%)	0.0342	0.0347(1.4%)	0.0342	0.0344(0.7%)
Extreme	Loss	0.0313	0.0315(0.7%)	0.0313	0.0316(1.0%)	0.0315	0.0318(1.2%)
2%	Absolute return	0.0308	0.0310(0.6%)	0.0308	0.0309(0.4%)	0.0305	0.0308(0.7%)
All cases	Loss	0.0092	0.0094(1.8%)	0.0092	0.0093(1.1%)	0.0091	0.0093(2.6%)
	Absolute return	0.0098	0.0100(2.2%)	0.0098	0.0100(2.0%)	0.0097	0.0099(1.9%)

(b) $w = m = 60$

Table 8: The RMSEs of the LASSO rolling-window prediction study for the cases $w = m = 30$ and $w = m = 60$.

S7 Appendix: Sensitivity analysis for the parameter m', the number of days of data included in the m'-day rolling window p-th percentile

The parameter m', the number of days of data included in the m'-day rolling window p-th percentile, affects the choice of the tail events. The choice of m' is flexible here; it depends on how we define "extreme". When we set a m', we want to filter out the cases that are extreme in the most recent m' days. Choosing a large m' often results in filtering a set of more extreme cases. Table 9a and Table 9b show the RMSEs of the LASSO prediction performance using $m' = 40$ and $m' = 60$, respectively. We can also observe improvements in RMSEs with the inclusion of the order distances as predictors using $m' = 40$ and $m' = 60$. Table 10a and Table 10b show the numbers of cases for which we accept the prediction under the tail-event strategy using $m' = 40$ and $m' = 60$, respectively. The numbers of days is larger overall when we choose a smaller m'. Hence, the proposed methodology is robust in regard to the parameter m', and the choice of m' can be determined by the person running the model.

Case	Response/Model	H_0	H_{1a}	H_0	H_{1b}	H_0	H_2
Extreme	Loss	0.0323	0.0332(2.8%)	0.0323	0.0316(**-2.2%**)	0.0319	0.0314(**-1.4%**)
0.1%	Absolute return	0.0295	0.0300(1.7%)	0.0295	0.0289(**-2.1%**)	0.0294	0.0291(**-1.0%**)
Extreme	Loss	0.031	0.0316(2.0%)	0.031	0.0303(**-2.3%**)	0.0306	0.0301(**-1.7%**)
1%	Absolute return	0.0298	0.0304(1.8%)	0.0298	0.0294(**-1.4%**)	0.0298	0.0296(**-0.4%**)
Extreme	Loss	0.0286	0.0292(1.8%)	0.0286	0.0280(**-2.1%**)	0.0285	0.0281(**-1.3%**)
2%	Absolute return	0.028	0.0284(1.3%)	0.028	0.0277(**-0.9%**)	0.028	0.0279(**-0.5%**)
All cases	Loss	0.0094	0.0097(3.7%)	0.0094	0.0098(4.7%)	0.0092	0.0097(5.8%)
	Absolute return	0.0099	0.0104(4.2%)	0.0099	0.0106(6.5%)	0.0097	0.0102(4.7%)

(a) $m' = 40$.

Case	Response/Model	H_0	H_{1a}	H_0	H_{1b}	H_0	H_2
Extreme	Loss	0.0352	0.0363(3.3%)	0.0352	0.0344(**-2.2%**)	0.0346	0.0342(**-1.2%**)
0.1%	Absolute return	0.035	0.0353(1.1%)	0.035	0.0340(**-2.7%**)	0.035	0.0347(**-0.7%**)
Extreme	Loss	0.0327	0.0334(2.2%)	0.0327	0.0320(**-2.1%**)	0.0325	0.0320(**-1.4%**)
1%	Absolute return	0.0325	0.0328(1.1%)	0.0325	0.0319(**-1.8%**)	0.0325	0.0323(**-0.6%**)
Extreme	Loss	0.0304	0.0310(1.9%)	0.0304	0.0298(**-2.1%**)	0.0303	0.0299(**-1.5%**)
2%	Absolute return	0.0295	0.0296(0.1%)	0.0295	0.0292(**-1.2%**)	0.0295	0.0291(**-1.2%**)
All cases	Loss	0.0094	0.0097(3.7%)	0.0094	0.0098(4.7%)	0.0092	0.0097(5.8%)
	Absolute return	0.0099	0.0104(4.2%)	0.0099	0.0106(6.5%)	0.0097	0.0102(4.7%)

(b) $m' = 60$.

Table 9: The RMSEs of the LASSO rolling-window prediction study using different m'.

	Response	Number of cases
Extreme 0.1%	Loss	84
	Absolute return	79
Extreme 1%	Loss	107
	Absolute return	105
Extreme 2%	Loss	139
	Absolute return	132

(a) $m' = 40$.

	Response	Number of cases
Extreme 0.1%	Loss	62
	Absolute return	55
Extreme 1%	Loss	90
	Absolute return	83
Extreme 2%	Loss	116
	Absolute return	113

(b) $m' = 60$.

Table 10: The number of trading days out of 3333 days for which we accept the predictions in the RMSE assessments with extremes of 0.1%, 1%, and 2% when using different values of m'. The rows indicate the responses (loss and absolute return) to predict.

S8 Appendix: An additional rolling-window prediction study using the Dow Jones Industrial Average Index

We have applied our proposed methodology using the historical data from the Hang Seng Index, and shown that the order distances can improve the prediction performances of the index under extreme scenarios. While the Hong Kong Stock Exchange is one of the most representative markets in the world [1], it is still necessary to test if the proposed methodology would be useful in other markets. We apply the same methodology to the Dow Jones Industrial Average (DJIA) Index. We obtain the closing prices of DJIA Index and its 30 constituent stocks from 19 September 2017 to 16 September 2022 (1,258 trading days). The details of the stocks are shown in Table 13 in the Support Information S9 Appendix. Then, we can obtain $T = 1,257$ daily returns from the daily prices. The parameters are set to be the same value as in the study using the HSI data in the paper, except we have provide the prediction performances using the maximum number of parents for each node $M = 7$ and $M = 13$ in order to perform sensitivity analysis. We learn the networks using $w = 30$ trading days of rolling-window data. A rolling-window LASSO regression prediction study is conducted using s sample size of $m = 40$, with a total of $T - (w + m) = 1257 - (30 + 40) = 1187$ days of predictions.

The RMSEs of the predictions using $M = 7$ and $M = 13$ are shown respectively in Table 11a and Table 11b. The model using both modified network density and order distance as predictors as predictors helps reduce the predictive RMSEs for $M = 7$, while the order distance alone is useful to improve the predictive performance for $M = 13$ in some cases, though the performance is not as good as that using $M = 7$. Table 12 shows the number of days for which we accept the predictions using a tail-event strategy. The number of cases ranges from 37 (3% of 1,187 trading days) to 57 days (4.8% of 1,187 trading days). The proportions are larger than those for the HSI data, where they range from 37 (1% of all 3,333 trading days) to 116 days (3.5% of 3,333) trading days, indicating that more tail events are detected for the DJIA Index than for the HSI using the same tail-event strategy.

As shown in the sensitivity analysis for the parameter M in the Supporting Information S4 Appendix, the choice of M indeed affects the results. The results using DJIA Index also show that a larger M may not be better, as opposed to the results in the Supporting Information S4 Appendix. Our proposed method provides the predictive performances for different M, to enable sensitivity analysis, which aims to show that the order distance can provide additional information to improve the predictive performances. Alternatively, we can also tune the parameter M on each trading day such that we can minimize some objective functions, for example, the predictive RMSEs in a training data set. Ensemble models using different M is also a possible method.

To conclude, we have shown that the proposed methodology using order distance as a predictor also improves the predictive performance for the DJIA Index. While the model improves the predictive performances for both the HSI and the DJIA Index, we need to be mindful of parameters tuning.

Case	Response/Model	H_0	H_{1a}	H_0	H_{1b}	H_0	H_2
Extreme	Loss	0.0408	0.0407(**-0.3%**)	0.0408	0.0412(0.8%)	0.041	0.0407(**-0.8%**)
0.1%	Absolute return	0.0302	0.0291(**-3.8%**)	0.0302	0.0311(2.9%)	0.0309	0.0290(**-6.0%**)
Extreme	Loss	0.0352	0.0357(1.3%)	0.0352	0.0354(0.4%)	0.0355	0.0354(**-0.5%**)
1%	Absolute return	0.0292	0.0275(**-5.6%**)	0.0292	0.0302(3.3%)	0.0293	0.0274(**-6.6%**)
Extreme	Loss	0.0322	0.0325(1.1%)	0.0322	0.0323(0.2%)	0.0324	0.0322(**-0.7%**)
2%	Absolute return	0.0264	0.0253(**-4.4%**)	0.0264	0.0272(2.8%)	0.0264	0.0249(**-5.5%**)
All cases	Loss	0.01	0.0100(0.1%)	0.01	0.0102(1.9%)	0.0092	0.0095(3.1%)
	Absolute return	0.0103	0.0105(2.1%)	0.0103	0.0106(2.7%)	0.0098	0.0101(2.5%)

(a) $M = 7$

Case	Response/Model	H_0	H_{1a}	H_0	H_{1b}	H_0	H_2
Extreme	Loss	0.0408	0.0437(7.0%)	0.0408	0.0425(4.2%)	0.0410	0.0434(5.8%)
0.1%	Absolute return	0.0302	0.0312(3.1%)	0.0302	0.0310(2.5%)	0.0309	0.0317(2.7%)
Extreme	Loss	0.0352	0.0374(6.2%)	0.0352	0.0363(3.2%)	0.0355	0.0371(4.4%)
1%	Absolute return	0.0292	0.0292(0.1%)	0.0292	0.0288(**-1.2%**)	0.0293	0.0294(0.3%)
Extreme	Loss	0.0322	0.0339(5.3%)	0.0322	0.0334(3.6%)	0.0324	0.0335(3.4%)
2%	Absolute return	0.0264	0.0268(1.3%)	0.0264	0.0259(**-2.2%**)	0.0264	0.0267(1.2%)
All cases	Loss	0.010	0.0106(5.6%)	0.01	0.0106(6.5%)	0.0092	0.0100(7.7%)
	Absolute return	0.0103	0.0112(9.1%)	0.0103	0.0102(**-1.1%**)	0.0098	0.0103(5.0%)

(b) $M = 13$

Table 11: The RMSEs of the LASSO rolling-window prediction study using different values of M for the DJIA Index data.

	Response	Number of cases
Extreme 0.1%	Loss	37
	Absolute return	37
Extreme 1%	Loss	47
	Absolute return	44
Extreme 2%	Loss	55
	Absolute return	57

Table 12: The number of trading days out of 1187 days for which we accept the predictions in the RMSE assessments with extremes of 0.1%, 1%, and 2%, using the DJIA Index data. The rows indicate the responses (loss and absolute return) to predict.

S9 Appendix: Details of the DJIA Index constituent stocks

The names and stock symbols of the 30 DJIA Index constituent stocks, sorted by name, are shown in Table 13.

Constituent Name	Stock symbol
3M	MMM
American Express	AXP
Amgen	AMGN
Apple	AAPL
Boeing	BA
Caterpillar	CAT
Chevron	CVX
Cisco	CSCO
Coca-Cola	KO
Disney	DIS
Dow	DOW
Goldman Sachs	GS
Home Depot	HD
Honeywell	HON
IBM	IBM
Intel	INTC
Johnson & Johnson	JNJ
JPMorgan Chase	JPM
McDonald's	MCD
Merck	MRK
Microsoft	MSFT
Nike	NKE
Procter & Gamble	PG
Salesforce	CRM
Travelers	TRV
UnitedHealth	UNH
Verizon	VZ
Visa	V
Walgreens Boots Alliance	WBA
Walmart	WMT

Table 13: The names and stock symbols of the DJIA Index constituent stocks, sorted by name.

References

1. Cattlin, B. What are the largest stock exchanges in the world? [cited 7 Oct 2022]. *IG.* https://www.ig.com/uk/trading-strategies/what-are-the-largest-stock-exchanges-in-the-world--180905.
